# “Comprehensive Healthcare for America”: Using the Insights of Behavioral Economics to Transform the U. S. Healthcare System

**DOI:** 10.1017/jme.2023.52

**Published:** 2023

**Authors:** Paul C. Sorum, Christopher Stein, Dale L. Moore

**Affiliations:** 1: ALBANY MEDICAL COLLEGE, ALBANY, NY, USA; 2: SIENA COLLEGE, LOUDONVILLE, NY, USA; 3: ALBANY LAW SCHOOL, ALBANY, NY, USA

**Keywords:** Healthcare Reform, Universal Healthcare, Healthcare Access, Healthcare Financing, Behavioral Economics, Libertarian Paternalism, Single Payer

## Abstract

“Comprehensive Healthcare for America” is a largely single-payer reform proposal that, by applying the insights of behavioral economics, may be able to rally patients and clinicians sufficiently to overcome the opposition of politicians and vested interests to providing all Americans with less complicated and less costly access to needed healthcare.

The United States needs an efficient, effective, and politically feasible way to provide all Americans with access to affordable healthcare. We propose a new healthcare system named “Comprehensive Healthcare for America” (CHA).

## The Need to Transform Our Healthcare System

The 2021 report of the Commonwealth Fund on the performance of the US healthcare system compared to those of 10 other high-income countries is damning. Even with the Affordable Care Act (ACA), the US ranks last in access to care, equity, administrative efficiency, and healthcare outcomes. As a result, it ranks far below all the others in overall performance, despite vastly higher spending.^1^ The American public agrees: in the West Health-Gallup survey in June 21-30, 2022, 44% gave the health system a grade of D or F.^2^ What can be done?

An obvious solution would be a single payer system, an expanded and improved “Medicare for All,” long advocated by Physicians for a National Health Program. It would provide universal access and comprehensive benefits, would be equitable, and would reduce administrative barriers and costs. The Medicare for All bills of Bernie Sanders in the Senate and Pramila Jayapal in the House of Representatives would, after a 2-4 year transitional buy-in period, institute automatic enrollment for everyone (except for those insured by the Department of Veterans Affairs and the Indian Health Service).^3^ This solution would be in line with the health systems of other high-income countries, which provide universal health insurance —generally considered a right — through either single payer systems or mixed systems with considerable governmental control.^4^

It is highly unlikely, however, that Congress will pass a Medicare for All bill. First, the Republican party now has a majority in the House of Representatives, and, even if the Democrats regain control, the Republicans would use the Senate filibuster to block any move toward single payer. Second, President Biden and many moderate Democrats are opposed to single payer. Instead, Biden campaigned in favor of a Medicare-like public option although in spring 2021 he backed away even from this.^5^ Third, single payer continues to be a difficult sell to voters, even though 67% of Republicans and 92% of Democrats agreed in January 2021 with “enacting federal legislation to ensure everyone has health insurance.”^6^ The Medicare for All bills would dramatically change healthcare financing, switching fully to federal taxes; would seem to many like a government take-over of healthcare; and would not allow people to opt out and keep the private insurances with which they are satisfied. But the majority of Americans are opposed to tax increases, are suspicious of the federal government, and like their current insurance plans.^7^ Even the 63% who agreed in a Pew survey in July-August 2020 that “it is the federal government’s responsibility to make sure all Americans have health care coverage” were divided into 36% who favored a single government program and 26% a mixture of government and private programs.^8^

We propose a plan that is as close as possible to Medicare for All, but that may be able to overcome the political obstacles to adoption: “Comprehensive Healthcare for America” (CHA). To explain it, we need to address seven questions. First, how can behavioral economics show us the path to take? Second, why are other proposals to achieve universal healthcare coverage inadequate? Third, what are the tenets of CHA and how does the plan differ from Medicare for All? Fourth, what impact will CHA have on healthcare expenditures? Fifth, why might CHA be able to rally sufficient support to achieve its adoption? Sixth, how will CHA be implemented? Seventh, what issues should be postponed until after CHA is implemented?

Must we resign ourselves to merely gradual and piecemeal changes? Health policy experts have envisioned many excellent reforms, most notably Micah Johnson and Donald Berwick’s “Medicare 2.0,” the transformation of traditional Medicare into a single plan with comprehensive benefits, and Ezekiel Emanuel and colleagues’ proposed extension to other states of Maryland’s use of global hospital budgets with equal payments to the hospitals by all payers, now known as the Maryland Total Cost of Care Model (TCCM).^9^ The hospitals in TCCM are motivated by the global budgets to increase savings by directing funds to outpatient providers and to underserved areas in order to improve the value of out-patient care and thereby reduce unneeded hospitalizations. Even if the successes in Maryland are real,^10^ however, extending this program to other states would be difficult and slow, as Emanuel and his colleagues recognize. Moreover, both Medicare 2.0 and TCCM would leave us with a complex system of multiple insurers and be unlikely to achieve universal coverage.

The best path to universal access may seem to be, therefore, to institute what the Yale political scientist Jacob Hacker labels “Public Option 2.0.”^11^ Hacker has long argued for the creation of the Medicare-based public option that was abandoned for political reasons first by President Obama, then by President Biden. “Public Option 2.0” would be part of Medicare, using its national network of providers and basing their reimbursement on Medicare rates. It would have no deductibles and would cover all primary care without copayments. It would automatically enroll low-income people without insurance and be available through the ACA marketplace to those not eligible for other public insurances and even to workers with employer-sponsored insurance. It would, however, allow private plans to be available in the ACA marketplace as well as from employers. Hacker argues that his plan is politically feasible, since most of the public supports a public option,^12^ and that it could then evolve into something close to Medicare for All. The increasing privatization of Medicare — through Medicare Advantage and recently through Direct Contracting Agencies and now the Accountable Care Organization Realizing Equity, Access, and Community Health (ACO REACH) model — threatens, however, to leave little traditional Medicare left to expand.^13^ More fundamental reform than a public option is, therefore, needed right away.

We propose a plan that is as close as possible to Medicare for All, but that may be able to overcome the political obstacles to adoption: “Comprehensive Healthcare for America” (CHA). To explain it, we need to address seven questions. First, how can behavioral economics show us the path to take? Second, why are other proposals to achieve universal healthcare coverage inadequate? Third, what are the tenets of CHA and how does the plan differ from Medicare for All? Fourth, what impact will CHA have on healthcare expenditures? Fifth, why might CHA be able to rally sufficient support to achieve its adoption? Sixth, how will CHA be implemented? Seventh, what issues should be postponed until after CHA is implemented?

## 1. Lessons of Behavioral Economics: Achieving Acceptance of CHA

Psychologists have demonstrated that people’s judgments and decisions can be greatly affected by how a choice is framed, by risk aversion, by inertia and status quo bias, and by the greater weighting of losses than of gains.^14^ Inertia and avoidance of complex choices have repeatedly prevented people from switching to more beneficial insurance plans.^15^ Accordingly, people who currently have private insurance would be unlikely to switch to a “public option,” even if they support it for other people, or to favor “single payer” if it means giving up their current insurance.

Behavioral economists can, however, show us the road to take. According to the “libertarian paternalism” proposed by Richard Thaler and Cass Sunstein, “people should be free to do what they like — and to opt out of undesirable arrangements if they want,” but “it is legitimate for choice architects to try to influence people’s behavior in order to make their lives longer, healthier, and better,” i.e., to “make choosers better off, *as judged by themselves*.”^16^ Every choice has a structure, a framework, even if it is only a starting point; the aim of the paternalist is to alter the structure, to reframe the choice enough to give people a “nudge.” An often-cited demonstration of choice architecture — in particular, the power of default options — is the much higher rate of organ donation when people must opt out if they do not want to donate after death than when people have to opt in.^17^

Automatic enrollment increases participation in retirement plans; indeed, the Pew Charitable Trusts found in 2018 that plans that auto-enrolled had participation rates exceeding 90 percent compared with rates in the 50 percent range for plans in which workers had to opt in.^18^ Moreover, the value of automatic enrollment in health insurance has been demonstrated by the successes not only of Medicare — in which enrollment in Parts A and B is automatic for those turning 65 who have opted to get Social Security payments — but also of a program called Express Lane Eligibility (ELE) for the Children’s Health Insurance Program (CHIP). In 2009, Congress allowed states to use ELE to provide the automatic enrollment into CHIP of children in financially qualified families. Although only 14 states adopted ELE, even temporarily, the results were higher enrollment with lower administrative costs.^19^ When Louisiana subsequently changed the enrollment process to require parents to check an opt-in box on the Supplemental Nutrition Assistance Program application form, ELE enrollment fell by 62%.^20^

Recently multiple experts have presented detailed plans to use automatic enrollment to reduce the number of persons without health insurance.^21^ Their proposals have, however, major flaws:

- They are partial, i.e., they apply automatic enrollment only to portions of the population, not all (even if the currently uninsured are the portion most in need).
- As a result, they maintain — and possibly increase — the complexity of the current health insurance system, with its multitude of different types of insurance.
- In consequence, they do not decrease the excess administrative expenses and the hassles to patients, physicians, and hospitals of the current system.
- Instituting them would involve a multiplicity of legal and administrative changes.
- The proposal of Linda Blumberg and colleagues, in particular, does not allow people to opt out of health insurance, i.e., requires them to pay for coverage (if sufficient income) whether they want it or not.^22^ It is, therefore, likely to elicit libertarian objections, thereby raising a further political obstacle to its adoption (as the authors acknowledge).

We can, nonetheless, conclude that, to achieve people’s buy-in, our plan needs to be simple and automatic for them, to avoid threatening what they like, to nudge them toward what they will see as benefits, and to allow them the freedom to opt out.

## 2. Proposals to Reform the Healthcare System

Two types of proposals to achieve universal access fall in between the dichotomy (seen, for example, by American College of Physicians in its 2020 position papers^23^) of a public option vs. Medicare for All. Each finds echoes in the health system of other wealthy nations:^24^

- The first is to put a central administration on top of our current multi-payer system to achieve universal coverage with greater uniformity and equity. This would bring us closer to the systems in the Netherlands, Switzerland, and Germany composed of multiple private insurance plans, although these plans are not-for-profit.
- The second type of reform is to establish an improved Medicare, with the coverage provisions of Medicare for All proposals, but with the continuation of Medicare Advantage and other privately-run insurance plans. Nations like Germany and even Canada and the United Kingdom allow some people to get their care from private insurance plans, i.e., have two-tier systems, but to a far less degree than in the US now or with these proposals.

### Creating a Central Administration

Three very different ways of putting a unifying central administration on top of a largely private system have been proposed. Each makes use of an existing administrative body, but they differ greatly in the power left to private insurance companies:

- The health policy commentator Stuart Butler of the Brookings Institute advocates “Medicare Advantage for All,” an expansion of the already increasing privatization of Medicare, based on managed care plans provided by private insurers who contract with Medicare (as also suggested recently by the Harvard physicians Greg Zahner, Peter Croughan, and Daniel Blumenthal).^25^
- The occupational health expert Nortin Hadler proposes the “Universal Workers’ Compensation Model,” an expansion of the workers’ compensation system to all the healthcare and other needs of employees.^26^ It would be administered by private insurance companies, but overseen by a Workers’ Compensation Commission, and covered benefits would be determined by an independent Clinical Effectiveness Panel. Although Hadler focuses on large employers at a state level (like current workers’ compensation), his plan could be expanded across the nation.
- The endocrinologist Richard Byyny, the executive director of the Alpha Omega Alpha medical honor society, argues instead — following the lead of former senator Tom Daschle and the Blue Ridge Academic Health Group in 2008 — for the creation of a “National Health Reserve System,” modeled on the US’s quasi-independent Federal Reserve System, that would regulate our system of private and governmental health insurance with the aim of providing high quality and cost-effective care for all.^27^

The major deficiency of these proposals is the perpetuation of a complex healthcare system with a multiplicity of private insurance companies.^28^

### Establishing an Improved Medicare

Two important plans presented in 2018-19 — “Medicare Extra for All” and “Medicare for America” — envision instead a greatly expanded and improved traditional Medicare, with at least some automatic enrollment, though short of single payer:

- The Center for American Progress’s “Medicare Extra for All” would increase current Medicare benefits and be available to all Americans. Newborns and individuals turning 65, the uninsured, and subsequently those in Medicaid and CHIP would be enrolled automatically.^29^
- HR 2452 “Medicare for America,” introduced into Congress in December 2018 and again in May 2019 by Representatives Rosa DeLauro and Jan Schakowsky, would go somewhat further in automatic enrollment than Medicare Extra for All by enrolling automatically not only the uninsured and those purchasing insurance on the individual market (including the ACA exchanges), but also those in Medicare, Medicaid, and CHIP.^30^

The Medicare Extra for All and Medicare for America plans have several deficiencies:

- They do not have automatic enrollment for large numbers of people, especially for those with employer plans; they ask these people to opt in, not opt out. Many people will, therefore, retain employer-sponsored insurance;^31^ the complexity of our current system will remain; and the problem of risk-selection — i.e., that private insurances will retain the healthy and privileged — will persist.^32^
- They retain Medicare Advantage (renamed Medicare Choice and Medicare Advantage for America, respectively), even though Medicare Advantage plans produce higher costs and cost-related problems for patients than traditional Medicare, but not better performance.^33^ Patients are attracted to Medicare Advantage currently, at least in part, because it is simpler to expand the inadequate coverage of traditional Medicare by enrolling in Medicare Advantage than by adding a Medigap plan and Medicare Part D drug coverage.^34^ But Medicare Extra for All and Medicare for America would have this expanded coverage. Accordingly, there would be no need, at least for most people, for Medigap and Part D and, therefore, no need for Medicare Advantage, even if its deficiencies are corrected (as envisioned in Medicare Extra for All and Medicare for America).
- Medicare Extra for All and Medicare for America maintain many copayments for the non-poor (even if Medicare for America gets rid of withholds). Yet surely any financial benefits to providers from collecting copayments and to the insurance system from incentivizing patients not to use care are reduced, if not eliminated, by the administrative burden on healthcare providers — and the resulting increase in the overall costs of care — of collecting co-payments^35^ and by the added harm and costs resulting from delaying needed care.
- As in the Medicare for All bills, many of the clinicians — though, admirably, not those in primary care or (in Medicare for America) mental and behavioral health — would see their reimbursement rates drop to, or close to, Medicare levels. The aim is, of course, to reduce healthcare costs. As a result, however, many of these clinicians would oppose Medicare Extra and Medicare for America when the support of providers is needed to rally their patients and to counterbalance the expected opposition of insurance companies and drug manufacturers.

## 3. The Tenets of “Comprehensive Healthcare for America”

1. *Enrollment will be universal and require minimal effort*. After a year or two of planning and preparation — not of slow transition as in the Medicare for All bills — the plan will be inaugurated all at once. On that day, and subsequently at birth, all Americans will be insured by CHA; it will instantly become their default health insurance plan. They will be able to obtain their individual identification numbers easily when they next receive medical care or if they go to the CHA website or a local office.

2. *Coverage will be comprehensive*. CHA will, like the Medicare for All bills, cover all needed health services and products, including dental care, and it will also cover telemedicine and institutional long-term care.^36^ All healthcare providers (except for the few who might refuse to participate) will be covered. Patients will be able to keep their doctors, will not face any out-of-network bills, will be covered across the nation, and will not give up any benefits (except for those not justified by the medical evidence).

3. *The rules governing the functioning of the system will be simple, uniform, transparent, evidence-based, and protected from political interference*. As in Medicare for All, both patients and providers will easily know what to expect, including what services and products are covered. The rules and policies regarding administration and functioning will be made by an agency, like the current Center for Medicare and Medicaid Services (CMS), that is advised and supervised by a body of representatives of stakeholders (in particular, physicians, hospitals, patients, and government). The agency will receive advice on payment rates from a body of experts like the current Medicare Payment Advisory Commission. Determinations of coverage will be made by independent boards of experts — as free as possible from political interference — similar to the current United States Preventive Services Task Force and Advisory Committee on Immunization Practices, the British National Institute for Health and Care Excellence, and Hadler’s Clinical Effectiveness Panel. On the regional and local levels, the system will be administered — like traditional Medicare now or most companies who self-insure — by groups of experts who will have contracts for particular regions of the country.

4. *Methods of financing will remain largely unchanged* (at least in the short term), unlike in the Medicare for All bills. People’s direct and indirect payments for their own current and future care and for that of others who need support will be seamlessly redirected to the CHA central healthcare fund (similar to the financing of Medicare for All proposed by Johnson, Kishore, and Berwick^37^). These include the federal, state, and county tax revenues used to support Medicaid; the payments to private insurance companies by people who are self-insured in or outside of the ACA marketplace; the payroll deductions and employer contributions used for employer-provided insurances (whether the employers are self-insured or utilize private insurance companies); the payroll deductions sent to the Medicare trust fund for future use; the deductions from Social Security checks to pay for Medicare or Medicare Advantage; and the federal tax revenues used to support Medicare Parts B and D and Medicare Advantage. A new, highly progressive federal income tax can meet any need for further revenue, but it is unlikely to be necessary (as discussed below).

5. *People will not have to pay more for their healthcare* (with rare exceptions) than they do now; indeed, as in Medicare for All, most will pay less. The ACA already has created a reasonable uniformity in people’s insurance benefits, requiring private insurances to cover a set of essential benefits and outlining three possible levels of benefits to be offered on the health exchanges. As a result, their insurance payments (as outlined above) will be largely unchanged, including zero premiums for those currently in, or eligible for, Medicaid and CHIP. Small equity adjustments will need to be made on the principle that the baseline charge for CHA, before social equity adjustments, should be the same for all (possibly by using current ACA marketplace gold-level plans as a benchmark, as suggested in the Medicare for America plan). They will not have any copayments or deductibles for needed, evidence-based care, including tests and medications. If patients want—and physicians want to prescribe—services and products not supported by evidence, these will not be prohibited but must be paid for by supplementary private insurance or out-of-pocket.

6. *Reimbursement rates will be set to ensure that most healthcare providers will see no decrease in their net revenues.* Currently private insurers pay considerably more to physicians, hospitals, and other healthcare providers than do Medicare and Medicaid. The analyses done by single-payer supporters suggest that Medicare for All, as in the House and Senate bills, would lead to higher, not lower, revenues for physicians and hospitals.^38^ Nonetheless, to ensure providers’ support of CHA, it must be made very clear to them that the reimbursement levels will be sufficiently high — higher than proposed in Medicare for All, Medicare Extra for All, and Medicare for America — that any decrease in direct payments to providers will be more than offset by the declines in their administrative costs as well as by the increased consumption of services resulting from the expansion of coverage (in line with what is argued by Chown and colleagues^39^). The support of physicians is, indeed, crucial for public acceptance of CHA because people continue to trust their physicians, even if less so than in the past,^40^ and because studies in behavioral economics demonstrate the importance of endorsement by a trusted authority.^41^ At the same time, private companies that insure those patients who opt out of the CHA will not be allowed to pay providers more than CHA, thereby avoiding an invidious imbalance in payments^42^ and minimizing the number of providers who choose to opt out of CHA.

7. *Private insurance companies will have reduced roles but will not be abolished*. The expertise and, in many cases, dedication to patient welfare of the employees of private insurance companies should be recognized and utilized. The companies will still have three roles. First, they will provide management services for the new system, under contract, as is now done for self-funded employers and for Medicare. They will, for example, verify the credentials of providers, manage their claims, and run CHA programs to improve the quality of care and the healthy behaviors of patients; but they will no longer, of course, establish lists of preferred providers or of covered services. Second, they will provide extra non-essential services, outside of CHA, as allowed in Medicare for All proposals and as performed currently (to a greater degree) by Medigap plans and by supplementary insurers in, for example, France.^43^ Third, unlike in Medicare for All, they will provide insurance to those who choose to opt out of the system. The greatly reduced roles of private insurances correspond to their roles in the universal healthcare plans of other wealthy countries.^44^

8. *Individuals will have complete freedom to opt out,* easily and without restrictions, unlike in the Medicare for All bills. It is important to respect Americans’ long history of self-reliance and opposition to outside restrictions, even against their interests, as long as this does not harm others. Their contributions will be redirected to the private insurance company of their choice, with any level of coverage they want, and they will even be able to forgo health insurance entirely. Similarly, to be consistent, providers must be free to opt out of CHA to provide care through private plans or their own concierge practices.

Tenets 7 and 8 raise the specter of a two-tier system, with wealthier patients paying extra for more rapid access to services and for higher quality physicians and facilities, as happens to some degree in other countries.^45^ US patients do not face the long wait times for specialty services that have plagued the United Kingdom and Canada, and the incentives for patients and physicians to opt out will be greatly reduced by the clear benefits of CHA to both groups (as discussed below). Nonetheless, it will be prudent, indeed essential, to add further barriers:

- As in Germany and some Canadian provinces,^46^ physicians and other providers will not be able to contract with both CHA and private insurers. Those in CHA will be able to take care of patients who have opted out — it will be important to provide everyone with the care they need — but these patients must self-pay at the established CHA rates and, if they have private insurance, seek reimbursement on their own.
- Private insurers will not be allowed to pay physicians and others who have opted out at higher rates for CHA-covered services. Clinicians will, therefore, have a financial incentive to leave CHA only if they provide services that are deemed by the CHA board of experts as not worthy of coverage but that are reimbursed by private insurance; this will require that the CHA board not be too narrow in its coverage decisions.

## 4. Healthcare Expenditures under CHA: Potential for Increased Costs Offset by Multiple Opportunities for Savings

Medicare for All and CHA would be likely to cost less overall than the current system. Yet cost predictions vary from more to less depending on what assumptions are used.^47^ The expected impact of CHA on overall healthcare expenditures is the projected difference between increased costs and increased savings (as for Medicare for All plans).

### Sources of Potential Increased Expenditures

- CHA is likely to increase the consumption of health products and services. The number of people insured (namely, all residents except for opt-outs) will be expanded, and all evidenced-based care will be covered. The elimination of copayments and deductibles is likely — as in the classic RAND experiment^48^ — to increase unneeded as well as needed care. The analyses by Gaffney and colleagues of the impact of health coverage expansion here and abroad, however, find little net increases in healthcare use.^49^
- Further short-term expenses will include the costs of planning and implementation and the subsidies to help private insurance employees as they transition.
- In addition, unlike in the Medicare for All bills, payments to physicians and other providers will not be reduced to (or close to) Medicare rates.

### Opportunities for Savings

- The greatest savings, for both CHA and Medicare for All, will come from the reduction in administrative and other insurance-related costs^50^ — i.e., the monies spent by insurers (including the profits of for-profit insurers) and by providers related to billing, to coding and risk adjustment, to collecting copayments, and to utilization management.^51^ Woolhandler and Himmelstein reported in 2019 that insurance overhead was projected to cost $301.4 billion, including $252 billion for private insurers — about 12% of their premiums — in contrast to overhead of 1.6% in Canada’s single payer system and 2.2% in traditional Medicare.^52^ Even if all contracts for private insurers were, somehow, standardized and simplified and thereby — as modeled by Scheinker and colleagues^53^ — reduced billing-related administrative costs as much as, or more than single payer, the multiplicity of insurances would continue to impose other costs and hassles, such as different drug formularies and utilization processes.
- The costs of pharmaceuticals and devices will be lowered — or at least prevented from soaring (as is happening now) — as a result of central bargaining power, along the lines suggested by Gaffney and Lexchin (as in other countries).^54^ Furthermore, the CHA central fund will not pay for expensive medications and procedures that are not justified by evidence (such as Biogen’s aducanumab^55^), thereby restraining the increase in their consumption and use. The standardized cost measurement facilitated by a unified health system will permit more accurate cost-effectiveness calculations, when permitted (as discussed in Section 7), and a further decrease in waste.^56^
- Healthcare will no longer be a growing field of for-profit investment. The excessive prices negotiated by some hospitals (among others) with different insurance companies, including for physician-administered drugs, will be reduced and standardized.^57^ The increase in purchases of not-for-profit hospitals, group practices, individual physician practices, and even hospices by private equity firms and other for-profit companies — which can lead to higher charges, increased volume of services, and greater provision of low value services — will likely be reversed.^58^ Private entities acting as profit-making intermediaries — including private insurance companies, direct contracting agencies or ACO REACH, and pharmacy benefit managers — will no longer be needed.^59^ Academic leaders will be less likely to become involved with the for-profit industry.^60^
- The different “quality” programs imposed by Medicare and private insurers — which currently are burdensome to clinicians and, in the case of programs like the Merit-based Incentive Payment System and Accountable Care Organizations (ACOs), have had minimal impact on quality or on cost to insurers^61^ even though CMS claims moderate success for ACOs (although not assessing their impact on clinicians)^62^ — can also be simplified and standardized.
- Individual patients — especially those who are poor or live in underserved areas — will be less inclined, or forced, to postpone needed care^63^ or to resort to expensive health care sites (in particular, emergency departments and hospitals) because they will have increased access to primary and outpatient care without the disincentives of copayments or deductibles.

The overall healthcare savings might be slightly less after CHA is instituted than for Medicare for All because of payments for people’s institutional long-term care and the continuation of private insurance plans for those — likely only a small percentage (as argued below) — who opt out. The healthcare costs of opt-outs would not, however, count as part of CHA itself. As in Medicare for All, payments would shift from administrative-related tasks to the actual care of patients.

## 5. Overcoming the Political Barriers to CHCA

The political barriers facing CHA — like those facing Medicare for All — are clearly huge:

- It will be opposed by groups benefiting financially from the current system, including for-profit insurance companies, pharmaceutical and medical device companies, private investors in healthcare, and for-profit hospitals and health systems. These groups were already at least partly responsible for Biden’s abandonment of his initial plan to create a public option and allow Medicare to negotiate drug prices.^64^
- It will also be opposed, whether on ideological grounds or for political gain, by opponents of “big government” and “socialism,” i.e., of any interference in the “free market.”
- Moreover, it will be difficult to overcome people’s fear of and resistance to change, their opposition to any increased taxes, their attachment to their current insurance plans, and their distrust of big government and Washington politicians — and thereby to get them to vote for politicians who support CHA.
- In addition, supporters of a pure single payer system will object to the loss of efficiency resulting from maintaining current financing mechanisms and, above all, to the risk that allowing patients and physicians to opt out will lead to an unequal, two-tier system (despite the measures, explained above, to prevent this).

Consequently, the task of convincing enough members of Congress, worried about reelection and dependent on donations, to pass such a fundamental reform seems daunting. The remedy is, as already explained, to rally the support of the public, of healthcare providers, and of employers by applying insights learned from behavioral economics.

CHA is designed to overcome the resistance to change of a public already inclined toward a universal healthcare system:^65^

- The transition will be quick and easy. People will be insured automatically. CHA will immediately become their default insurance option, their status quo. They will be able to obtain their insurance identification numbers the next time they need medical care.
- CHA will not raise taxes, instead keeping the financing mechanisms essentially unchanged.
- It will make minimal changes in how people get healthcare. People will see immediately that they will have no losses in terms of coverage and providers, only gains, i.e., that they may lose their insurer, but not their insurance, and that they can keep their doctors.
- People will quickly realize that they no longer face the increasing healthcare-related financial threats, namely the escalating drug prices, surprise billing, rising premiums and co-payments, high deductibles, and increase in other out-of-pocket costs.^66^ Indeed, lowering drug prices was rated by Americans in January 2021 as their second highest priority for President Biden and the new Congress.^67^ People will no longer be dissuaded by these costs from taking their prescribed drugs and from seeking care for important problems or for prevention.^68^ While in June 2020, an estimated 17.8% of individuals had medical debt, at a mean amount of $429, under CHA they will no longer fall, or fear falling, into medical debt.^69^
- CHA will reduce the current burden on patients of their own administrative tasks, such as interacting with their health benefits administrators, getting information from insurance companies, resolving billing issues, and coping with “phantom networks” when trying to access care.^70^
- It will allow people who want to keep their private insurance or who are opposed to what they see as government control to opt out of CHA or of any insurance.

The design will also rally physicians, who increasingly favor Medicare for All, even within the American Medical Association, but who often worry that a single payer system will negatively affect their professional autonomy, their workloads, and their incomes:^71^

- It will be clear to physicians, other clinicians, and their staffs that CHA will simplify the rules and procedures and will reduce the distracting and costly burdens imposed by the multiplicity of payers. For example, surveys in 2006 in the US and Canada found that physician practices in the US spent $87, 975 per physician per year interacting with payers, in contrast to only $22,205 in Ontario, Canada.^72^
- Clinicians, who are currently facing the threat of reduced reimbursements from Medicare,^73^ will also be assured that their net incomes will not be reduced.
- Their representatives will have a key role in managing the system (including, as experts, in deciding on coverage).
- They can expect a decrease in the burden of documentation in the electronic health record as its purpose shifts from justifying the level of billing to recording what is needed for patients.^74^
- A central administration will be able to remedy the lack of interoperability of electronic health records that impairs clinicians’ ability to provide optimal care.^75^
- Moreover, CHA will reverse the increasing for-profit orientation of American medical practice (especially with the intrusion of private equity firms).^76^ It will stop the erosion of the patient-physician relationship that is at the heart of clinical care and will allow physicians to refocus on the ethic of service to patients.^77^

Physicians are likely, therefore, to become outspoken advocates of CHA. As explained in Tenet 6, since people tend to trust their physicians, their endorsement will, in accordance with behavioral economics, have the important secondary effect of increasing public support.

Employers too will see the benefits of CHA.

- Although they will still need to send payroll deductions to CHA, they or their Human Resources departments will no longer have the burden of choosing what insurance plans to offer to their employees and signing them up.
- Individual employers and employer groups will not have to worry about the rising healthcare prices affecting their employees’ health and morale. Efforts of these groups to restrain these prices have been mostly unsuccessful.^78^

## 6. The Implementation Process

Once the political barriers have been overcome, the implementation of CHA will take lots of work — like any fundamental reform of a vast and complicated system — but it will not be as difficult as it might seem:

- The key parts of the healthcare system — the delivery of care by physicians and other healthcare professionals and by hospitals and other organizations — will be largely unaffected, except to become simpler and more seamless.
- The switch in insurance coverage will be automatic, and enrollment will require minimal effort by patients. Starting on the day of inauguration, signing up will be simple. All residents — though Congress may insist on restricting this to legal residents — will need only to show proof of identity at clinical sites (doctors’ offices, urgent care centers, emergency departments, hospitals) or at the CHA website or local offices to obtain their CHA identification numbers. These numbers will be needed when patients obtain care to ensure the integrity of the medical record, i.e., to make sure that each healthcare episode is connected to the correct patient record, providing current and future providers with accurate information. Congress may want to simplify the process by linking the new CHA numbers with the Social Security numbers that most people have already and that parents can request for their newborns before they leave the hospital.
- The financing of the system will change little, at least at first. As explained in Tenet #5, current payments for all types of insurance (other than the uniformed services and the Indian Health Service) will be redirected quite easily to the CHA central fund. Clearly this system, once established, will need to evolve quickly to make it as fair and equitable as possible.
- The basis of the central administration already exists in CMS and its advisory bodies. Their composition and roles would need to be altered and expanded, and the new committees of experts on coverage would need to be appointed and to decide on the goods and services to be reimbursed.
- Although the manner of administering the system on the local and regional levels — the most difficult aspect — will need to be set up in advance, the expertise already exists in the private companies used by Medicare as regional administrators; in the private insurance companies who, whether acting for themselves or as administrators for self-insured companies, are proficient in such necessary tasks as verifying the credentials of health care providers, insisting on high quality of care, and reimbursing physicians and others for their services (whether by fee-for-service or by capitation); and in the specialized firms that service self-insured companies. Working out the details between the CHA administrative board and these private companies and agreeing on specific contracts must be completed before inaugurating CHA. The preparation for the inauguration of CHA will, therefore, be complicated, will involve difficult negotiations with a variety of current stakeholders, and may take up to 2 years (the transition period envisioned in Jayapal’s Medicare for All bill). To facilitate this, the planning group for CHA will need to recognize the good intentions and know-how of most actors in the current system, integrate these people into CHA insofar as feasible, pay the costs of transition and reimburse outmoded entities for their short-term financial losses, and help those who do not become part of CHA to train for and find new positions.

## 7. Issues to be Addressed after Implementation

To keep the focus on the key elements of CHA, many important issues can, and should, wait to be resolved until after CHA has been inaugurated (unlike the ACA with its 906 pages^79^). These issues are, to varying degrees, complicated and controversial and risk distracting too much from the main task of implementing CHA as smoothly and rapidly as possible.

- **Finance:** whether and how payments made by individuals — initially based on their current contributions or pegged to gold-level ACA policies — need to be adjusted to maximize fairness, and, indeed, whether financing should be shifted from payroll deductions to graduated federal income taxes (which might simplify the collection of funds).
- **Role of cost in coverage decisions:** whether and how CHA (in particular, its board of experts) should consider the costs of medications, devices, and procedures — in addition to their effectiveness — in making decisions on coverage. This is politically contentious. Fearing the charge of rationing, Medicare has long avoided using cost-effectiveness analysis (CEA) to determine the coverage of treatments (although it has used CEA to decide on preventive measures).^80^ The ACA created the Patient-Centered Research Institute but prohibited it from using cost per quality-adjusted life year thresholds “(or similar measure that discounts the value of a life because of an individual’s disability)”^81^ — it was to focus on comparative effectiveness, not cost effectiveness.^82^ The ACA also created the Independent Payment Advisory Board (IPAB) with the task of controlling Medicare costs without rationing care.^83^ The Obama administration did not, however, set up the IPAB because of the opposition of medical professionals (who realized that the only way it could cut costs would be to reduce reimbursements) and Republicans (who called it a “death panel”) and because of a temporary stabilization of Medicare spending.^84^ Congress repealed it in 2018.
- **Payment for healthcare delivery:** whether and to what extent, in the new context of CHA, reimbursement of clinicians should continue to be, at least partly, by fee-for-service — noting that in France, Germany, and Japan, payment levels are the product of structured negotiations with physician associations^85^ — or should move away completely from fee-for-service;^86^ and whether hospitals should be financed via global budgets, as done in several other countries, as proposed in Medicare for All plans, and as tested in Maryland’s All-Payer Model.^87^
- **Value-based care:** how to promote quality of care in ways that are less burdensome on providers and more effective than those measures and methods used currently (thereby turning Donald Berwick’s Triple Aim into the Quadruple Aim).^88^
- **Electronic health records (EHRs):** how to integrate the multiple different EHRs used by clinicians and health systems throughout the country so that patients can allow clinicians anywhere in the US to access their health records.
- **Primary care:** how best to promote primary care both in underserved areas and in general, given the dwindling number of primary care physicians, the increasing use of urgent care centers, the offering of more telemedicine by third parties (such as Amazon), and the increasing control of primary care practices by for-profit companies.^89^
- **Further extension of CHA:** whether and how to integrate the Veterans Health Administration and TRICARE — which like Medicare are being increasingly privatized^90^ — and the Indian Health Service into the CHA, and whether to include noncitizen immigrants. Including noncitizens would be beneficial to society by keeping them as healthy and productive as possible and preventing a recourse to expensive emergency care.^91^
- **Health system consolidation:** how to respond to the newly dominant role in care delivery of health systems — which the majority of physicians now contract with, are integrated into, or are employed by — and to the increasing purchase of these systems and other aspects of healthcare by for-profit entities. The leaders of Physicians for a National Health Program propose a transition of health systems to “public, community-based ownership” and “an orderly conversion of investor-owned, for-profit providers to not-for-profit status.”^92^
- **Medical education:** how to promote equity in medical education, recognizing the persistent underrepresentation of disadvantaged groups and Medicare’s key role currently in financing graduate medical training.^93^

## Conclusion

Large shifts in public opinion can lead to dramatic changes in public policies and laws: witness the widespread acceptance of gay rights, the legalization of same-sex marriage, and the allowance in most states of the adoption of children by same-sex couples. Yet other proposals with wide public support — such as gun control and combat of climate change — are stymied by the opposition of politically powerful special interests. Major healthcare reform may seem to be irrevocably, and increasingly, in the latter category, especially as private financial interests take greater control. Nonetheless, by adding features of “libertarian paternalism”^94^ — in particular, automatic enrollment, minimal added effort, little apparent change in how to get care, no increase in taxes, and the right to opt out with minimal hassle — this revision of Medicare for All may be able to rally enough patients, clinicians, and voters to overcome the opposition mounted by the insurance and pharmaceutical industries and other for-profit investors in healthcare. It is not impossible that, with COVID-19 and Black Lives Matter revealing the deficiencies and inequalities in the US healthcare system — including the precarity of employer-based health insurance^95^ — Americans may finally be ready to undertake fundamental reform^96^ and, with CHA, to bring the American healthcare system up to, and beyond, the level attained by other wealthy countries.

## Note

The authors have no conflicts to disclose.

## References

[1] E.C. Schneider, A. Shah, D.M. Doty, R. Tikkanen, K. Fields, and R.D. Williams II, “Mirror, Mirror 2021 - Reflecting Poorly: Health Care in the U.S. Compared to Other High-Income Countries,” *Commonwealth Fund*, August 2021*, available at* <https://www.commonwealthfund.org/publications/fund-reports/2021/aug/mirror-mirror-2021-reflecting-poorly> (last visited January 1, 2022); E.Wager, J. Ortaliza, and C. Cox, “How does Health Spending in the U.S. Compare to other Countries?” *Kaiser Family Foundation*, January 21, 2022, *available at* <https://www.healthsystemtracker.org/chart-collection/health-spending-u-s-compare-countries-2/#GDP%20per%20capita%20and%20health%20consumption%20spending%20per%20capita,%202020%20(U.S.%20dollars,%20PPP%20adjusted)> (last visited August 1, 2022); M.Z. Gunja, E.D. Gumas, and R.D. Williams II, “Are Financial Barriers Affecting the Health Care Habits of American Men? A Comparison of Health Care Use, Affordability, and Outcomes Among Men in the U.S. and Other High-Income Countries,” *Commonwealth Fund Issue Brief*, July 14, 2022, *available at* <https://www.commonwealthfund.org/publications/issue-briefs/2022/jul/are-financial-barriers-affecting-health-care-habits-american-men> (last visited August 1, 2022); D.C. Radley, R.D. Williams II, M.Z. Gunja, J.C. Baumgartner, and E.D. Gumas, “Americans, No Matter the State They Live In, Die Younger Than People in Many Other Countries,” *Commonwealth Fund To the Poin*t, August 11, 2022, *available at* < 10.26099/8x1n-xg40> (last visited August 19, 2022).

[2] West Health-Gallup, “2022 HEALTHCARE IN AMERICA REPORT: America’s Report Card on the U.S. Healthcare System,” *available at* <https://s8637.pcdn.co/wp-content/uploads/2022/10/2022-Healthcare-in-America.pdf> (last visited December 2, 2022).

[3] Public Citizen, “The Case for Single Payer,” Report, February 4, 2019, *available at* <https://www.citizen.org/wp-content/uploads/migration/the_case_for_medicare-for-all_-_february.pdf> (last visited January 1, 2022); S. Woolhandler and D. Himmelstein , “Single-Payer Reform—‘Medicare for All,’” JAMA 321, no. 24 (2019): 2399–2400; P.S. Arno and P. Caper, “Medicare for All: The Social Transformation of US Health Care,” *Health Affairs Blog*, March 25, 2020, *available at* <https://www.healthaffairs.org/do/10.1377/hblog20200319.920962/full/> (last visited January 1, 2022); P. Jayapal, “Medicare for All Act of 2021,” H.R.1976, 117^th^ Congress, March 17, 2021, *available at* <https://www.congress.gov/bill/117th-congress/house-bill/1976/text> (last visited July 9, 2022); B. Sanders, “Medicare for All Act of 2022,” 117^th^ Congress, 2^nd^ Session, *available at* <https://www.sanders.senate.gov/wp-content/uploads/Medicare-for-All-2022-Bill.pdf> (last visited July 5, 2022). 31150046 10.1001/jama.2019.7031

[4] S.A. Glied, M. Black, W. Lauerman, and S. Snowden, “Considering “Single Payer” Proposals in the U.S.: Lessons from Abroad,” *Commonwealth Fund Issue Brief*, April 11, 2019, *available at* <https://www.commonwealthfund.org/publications/2019/apr/considering-single-payer-proposals-lessons-from-abroad> (last visited January 1, 2022); R. Tikkanen, R. Osborn, E. Mossialos, A. Djordjevic, and G. Wharton G, eds, “International Profiles of Health Care Systems,” *Commonwealth Fund*, December 2020, *available at* <https://www.commonwealthfund.org/sites/default/files/2020-12/International_Profiles_of_Health_Care_Systems_Dec2020.pdf> (last visited January 1, 2022). 30990595

[5] Biden Harris Democrats, “Healthcare,” *available a*t <https://joebiden.com/healthcare/> (last visited January 1, 2022); D. Scott, “Why Democrats’ Ambitions for Health Care are Shrinking Rapidly,” *Vox*, May 7, 2021, *available at* <https://www.vox.com/policy-and-politics/22422793/joe-biden-health-care-plan-obamacare-medicare-public-option> (last visited January 1, 2022).

[6] A.B. Frakt and J. Oberlander , “Challenges to Medicare for All Remain Daunting,” Health Affairs 39, no. 1 (2021): 142–5; J.S. Hacker, “Between the Waves: Building Power for a Public Option,” *Journal of Health Politics, Policy and Law* 46, no. 4 (2021): 535-547; POLITICO and Harvard T.H. Chan School of Public Health, “The American Public’s Priorities for the New President and Congress,” January 2021*, available at* <https://cdn1.sph.harvard.edu/wp-content/uploads/sites/94/2021/01/Politico-HSPH-Jan-2021-PollReport.pdf> (last visited January 1, 2022). 10.1215/03616878-8970739 33493271

[7] See Frakt and Oberlander and Hacker, *supra* note 6; Kaiser Family Foundation, “Public Opinion on Single Payer, National Health Plans, and Expanding Access to Medicare Coverage,” October 16, 2020*, available at* <https://www.kff.org/slideshow/public-opinion-on-single-payer-national-health-plans-and-expanding-access-to-medicare-coverage/> (last visited January 2022); K. Baicker and A. Chandra, “What Values and Priorities Mean for Health Reform,” *New England Journal of Medicine* 383, no. 15: e89, *available at* < https://www.nejm.org/doi/full/10.1056/NEJMp2025966> (last visited January 1, 2022). 10.1056/NEJMp2025966 32937060

[8] B. Jones, “Increasing Share of Americans Favor a Single Government Program to Provide Health Care Coverage,” *Pew Research Center*, Fact Tank. September 29, 2020, *available at* <https://www.pewresearch.org/fact-tank/2020/09/29/increasing-share-of-americans-favor-a-single-government-program-to-provide-health-care-coverage/> (last visited January 1, 2022); R.J. Blendon and J.M. Benson, “The Implications of the 2022 Elections for Health Policy,” *New England of Journal Medicine* online, December 14, 2022, *available at* <https://www.nejm.org/doi/full/10.1056/NEJMsr2214949?query=featured_home> (last visited December 16, 2022).

[9] M. Johnson and D.M. Berwick , “Medicare 2.0 — A Vision for the Future of America’s Health Insurance Plan,” JAMA 328, no. 21 (2022): 2107–2108; E.J. Emanuel, D.W. Johnson, M. Guido, and M. Goozner, “Meaningful Value-Based Payment Reform, Part 1: Maryland Leads The Way,” *Health Affairs Forefront*, February 9, 2022*, available at* <https://www.healthaffairs.org/do/10.1377/forefront.20220205.211264 > (last visited July 8, 2022); “Meaningful Value-Based Reform, Part 2: Expanding The Maryland Model To Other States,” *Health Affairs Forefront*, February 10, 2022, *available at* <https://www.healthaffairs.org/do/10.1377/forefront.20220207.85767> (last visited July 8, 2022). 36409483 10.1001/jama.2022.22092

[10] T.A. Brennan, “Maryland Hospital All-Payer Model: Can It Be Emulated?” *Health Affairs Forefront*, May 31, 2022, *available a*t <https://www.healthaffairs.org/do/10.1377/forefront.20220526.939479> (last visited July 7, 2022).

[11] Hacker, *supra* note 6; J.S. Hacker , “Medicare for More — Why We Still Need a Public Option and How to Get There,” New England Journal of Medicine 385, no. 12 (2021): 1060–1062; C. Monahan and K. Lucia, “Congressional Proposals for a Federal Public Health Insurance Option,” *Commonwealth Fund Blog*, November 3, 2022, *available at* <https://www.commonwealthfund.org/blog/2022/congressional-proposals-federal-public-health-insurance-option> (last visited December 3, 2022). 34506090 10.1056/NEJMp2111494

[12] E. Winter and J.S. Hacker, “Voters Support a Public Option for Health Insurance,” Data for Progress, October 2020, *available at* <https://www.filesforprogress.org/memos/a-public-option-for-health-insurance.pdf> (last visited January 1, 2022); Kaiser Family Foundation, “KFF Health Tracking Poll - May 2021: Prescription Drug Prices Top Public’s Care Priorities,” June 3, 2021, *available at* <https://www.kff.org/health-costs/poll-finding/kff-health-tracking-poll-may-2021/> (last visited January 1, 2022).

[13] J. Kahn. “CMS Direct Contracting Scheme Will Privatize Medicare,” *Health Justice Monitor*, July 9, 2021, *available at* <http://healthjusticemonitor.org/2021/07/09/cms-direct-contracting-scheme-will-privatize-medicare/> (last visited January 1, 2022); R. Gilfillan and D.M. Berwick, “Medicare Advantage, Direct Contracting, and the Medicare ‘Money Machine,’ Part 2: Building on the ACO Model,” *Health Affairs Blo*g, September 30, 2021, *available a*t <https://www.healthaffairs.org/do/10.1377/forefront.20210928.795755/full/> (last visited January 1, 2022); J. M. McWilliams, “Don’t Look Up? Medicare Advantage’s Trajectory and the Future of Medicare,” *Health Affairs Forefront*, March 24, 2022, *available at* <https://www.healthaffairs.org/do/10.1377/forefront.20220323.773602> (last visited July 5, 2022); R. Gilfillan and D.M. Berwick, “The Emperor Still Has No Clothes: A Response To Halvorson And Crane,” *Health Affairs Forefront*, June 6, 2022, *available at* <https://www.healthaffairs.org/do/10.1377/forefront.20220602.413644> (last visited July 7, 2022); G.A Jacobson and D. Blumenthal , “Medicare Advantage Enrollment Growth: Implications for the US Health Care System,” JAMA 327, no. 24 (2022): 2393–2394; M. Freed, J. Fuglesten Biniek, A. Damico, and T. Neuman, “Medicare Advantage in 2022: Enrollment Update and Key Trends,” *Kaiser Family Foundation Issue Brief*, August 25, 2022, *available at* <https://www.kff.org/medicare/issue-brief/medicare-advantage-in-2022-enrollment-update-and-key-trends/> (last visited December 2, 2022).35604657

[14] D. Kahneman , Thinking Fast and Slow (New York: Farrar, Straus and Giroux, 2011); J. Jachimowicz, S. Duncan, E. Weber, and E. Johnson, “When and Why Defaults Influence Decisions: A Meta-Analysis of Default Effects,” *Behavioural Public Policy* 3, no. 2 (2019): 159-186.

[15] J.M. McWillaims , C.C. Afendulis , T.G. McGuire , and B.E. Landon , “Cognitive Functioning and Choice between Traditional Medicare and Medicare Advantage,” Health Affairs 30, no. 9 (2011): 1786–94; C.J. Lako, P. Rosenau, and C. Daw. “Switching Health Insurance Plans: Results from a Health Survey,” *Health Care Analysis* 19, no. 4 (2011): 312-28; E.J. Johnson, R. Hassin, T. Baker, A.T. Bajger, and G. Treuer, “Can Consumers Make Affordable Care Affordable? The Value of Choice Architecture,” *PLoS ONE* 8, no. 12 (2013): e81521, *available at* < 10.1371/journal.pone.0081521> (last visited January 1, 2022); S. Bhargava, G. Loewenstein, and J. Sydnor, “Do Individuals Make Sensible Health Insurance Decisions?” *National Bureau of Economic Research*, working paper 21160, May 2015, *available at* <www.nber.org/papers/w21160> (last visited January 1, 2022); S. Bhargava and G. Loewenstein, “Choosing a Health Insurance Plan: Complexity and Consequences,” *JAMA* 314, no. 23 (2015): 2505-2506; A. Feher and I. Menashe, “Using Email and Letters to Reduce Choice Errors among Market Enrollees,” *Health Affairs* 40, no. 5 (2021): 812-9. 21852301 PMC3513347

[16] R.H. Thaler and C.R. Sunstein , Nudge: Improving Decisions about Health, Wealth, and Happiness (New York: Penguin Books, 2009): quotation at 5.

[17] E.J. Johnson and D. Goldstein , “Medicine. Do Defaults Save Lives?” Science 302, no. 5649 (2003): 1338–1339; Thaler and Sunstein, *supra* note 16: at 177-84. 14631022 10.1126/science.1091721

[18] B.C. Madrian and D.F. Shea DF, “The Power of Suggestion: Inertia in 401(K) Participation and Savings Behavior,” *National Bureau of Economic Research*, working paper 7682, May 2000, *available at* <http://www.nber.org/papers/w7682> (last visited January 1, 2022); J. Scott and A. Blevins, “Automatic Enrollment Can Boost Retirement Plan Participation,” *Pew Charitable Trusts*, August 15, 2018, *available at* <https://www.pewtrusts.org/en/research-and-analysis/articles/2018/08/15/automatic-enrollment-can-boost-retirement-plan-participation> (last visited January 1, 2022).

[19] S. Hoag, A. Swinburn, S. Orzoi, M. Barna, M. Colby, B. Natze, C. Trenholm, F. Blavin, G.M. Kenney, and M. Huntress, “CHIPRA Mandated Evaluation of Express Lane Eligibility: Final Findings,” *Mathematica Policy Research & Urban Institute*, December 30, 2013, a*vailable a*t <https://www.mathematica.org/publications/chipra-mandated-evaluation-of-express-lane-eligibility-final-findings> (last visited January 1, 2022); S. Dorn S, M. Wilkison, and S. Benatar, “CHIPRA Express Lane Eligibility Evaluation: Case Study of Louisiana’s Express Lane Eligibility, Final Report,” *Mathematica Policy Research*, January 12, 2014, *available at* <https://www.urban.org/sites/default/files/publication/33691/413272-CHIPRA-Express-Lane-Eligibility-Evaluation-Case-Study-of-Louisiana-s-Express-Lane-Eligibility.PDF> (last visited January 1, 2022); P. Shafer and A.B. Frakt , “To Truly Build the Affordable Care Act into Universal Coverage, More Creativity Is Needed,” JAMA Health Forum 1, no. 10 (2020): e201234, *available at* <https://jamanetwork.com/journals/jama-health-forum/fullarticle/2771413> (last visited January 1, 2022). 36218543 10.1001/jamahealthforum.2020.1234

[20] S. Dorn, J.C. Capretta, and L.I. Chen, “Making Health Insurance Enrollment as Automatic as Possible,” *Health Affairs Blog,* Part 1, May 2, 2018, *available at* <https://www.healthaffairs.org/do/10.1377/hblog20180501.141197/full/> (last visited March 3, 2023), Part 2, May 3, 2018, *available at* <https://www.healthaffairs.org/do/10.1377/hblog20180501.219130/full/> (last visited March 3, 2023).

[21] Dorn et al., supra note 19; C.L. Young and S. Lee, “How Well Could Tax-Based Auto-Enrollment Work?” *USC-Brookings Schaeffer Initiative for Health Policy*, April 2020, *available at* <https://www.brookings.edu/research/how-well-could-tax-based-auto-enrollment-work/> (last visited January 1, 2022); L.J. Blumberg, J. Holahan, and J. Levitis, “How Auto-Enrollment Can Achieve Near-Universal Coverage: Policy and Implementation Issues,” *Commonwealth Fund Report*, June 2021, *available at* <https://www.commonwealthfund.org/sites/default/files/2021-06/Blumberg_how_auto_enrollment_can_achieve_near_universal_coverage_r.pdf> (last visited January 1, 2022); A. McIntyre and M. Shepard , “Automatic Insurance Policies — Important Tools for Preventing Coverage Loss,” New England Journal of Medicine 386, no. 5 (2022): 408–411. 35089664 10.1056/NEJMp2114189 PMC9597888

[22] Blumberg et al., *supra* note 21.

[23] R. Crowley , H. Daniel , T.G Cooney , and L.S. Engel , “Envisioning a Better U.S. Health Care System for All: Coverage and Cost of Care,” Annals of Internal Medicine 172, no. 2 (2020): S7–32. 31958805 10.7326/M19-2415

[24] Glied et al. and Tikkanen et al., *supra* note 4; C.M. Flood and B. Thomas , eds., Is Two-Tier Health Care the Future? (Ottawa: University of Ottawa Press, 2020).

[25] S.M. Butler , “Medicare Advantage for All, Perhaps?” JAMA Health Forum 1, no. 7 (2020): e200967, *available a*t <https://jamanetwork.com/journals/jama-health-forum/fullarticle/2769097> (last visited January 1, 2022); S.M. Butler, “Achieving an Equitable National Health System for America,” *Brookings Report*, December 9, 2020, *available at* <https://www.brookings.edu/research/achieving-an-equitable-national-health-system-for-america/> (last visited on January 1, 2022); G.J. Zahner, P.W. Croughan, and D.M. Blumenthal. “Medicare Advantage for All: A Potential Path to Universal Coverage,” *JAMA* 327, no. 1 (2022): 29-30, *available at* <https://jamanetwork.com/journals/jama/fullarticle/2787446> (last visited January 1, 2022).36218703

[26] N.M. Hadler , By the Bedside of the Patient: Lessons for the Twenty-First-Century Physician (Chapel Hill, NC: University of North Carolina Press, 2016): at 159–176; N.M. Hadler and S.P. Carter, *Promoting Worker Health: A New Approach to Employee Benefits in the Twenty-First Century* (Chapel Hill, NC: University of North Carolina Press, 2018).

[27] Blue Ridge Academic Health Group, “A United States Health Board,” Policy Proposal, Fall 2008, *available at* <http://whsc.emory.edu/blueridge/publications/archive/blue_ridge_policy_proposal_final.pdf> (last visited January 1, 2022); R.I. Byyny, “All Things Considered…The Future of the U.S. Health Care ‘System’,” *Pharos*, Summer 2020: 3-10, *available at* <https://www.alphaomegaalpha.org/wp-content/uploads/2021/03/2020_Summer_editorial.pdf> (last visited January 1, 2022); R.L. Byyby, “Now Is the Time to Enact a U.S. Health Care System,” *Pharos,* Spring 2021: 2-7, *available at* <https://www.alphaomegaalpha.org/wp-content/uploads/2021/05/2021_Spring_Editorial.pdf> (last visited January 1, 2022).

[28] J. Kahn, “Medicare Advantage for All: a Bad Idea for Single Payer,” *Health Justice Monitor*, December 22, 2021, *available a*t <http://healthjusticemonitor.org/2021/12/22/medicare-advantage-for-all-a-bad-idea-for-single-payer/> (last visited January 1, 2022).

[29] Center for American Progress, “Medicare Extra for All,” February 22, 2018, *available at* <https://www.americanprogress.org/issues/healthcare/reports/2018/02/22/447095/medicare-extra-for-all/> (last visited January 1, 2022).

[30] R. DeLauro, “Medicare for America Act of 2019 Summary,” *available at* <https://delauro.house.gov/sites/delauro.house.gov/files/Medicare%20for%20America%20of%202019%20Summary.pdf> (last visited January 1, 2022); Congress.Gov, “H.R.2452 – Medicare for America Act of 2019,” *available at* <https://www.congress.gov/bill/116th-congress/house-bill/2452/text> (last visited January 1, 2022).

[31] A. Saavoss, L. Koenig, B. Demiralp, J. Nair, and J. Sherif, “The Impact of Medicare for America on the Employer Market and Health Spending,” *KNG Health Consulting*, October 22, 2019, *available at* <https://americashealthcarefuture.org/wp-content/uploads/2019/10/KNG-Health-The-Impact-of-Medicare-for-America-FINAL-Report-Oct-2019-1.pdf> (last visited January 1, 2022).

[32] C.S. Ho, “Medicare for All and Medicare for America: What Are We Fighting Over? Part 1,” *Law and Political Economy Project Blog*, July 23, 2019, *available at* <https://lpeproject.org/blog/medicare-for-all-and-medicare-for-america-what-are-we-fighting-over-2/> (last visited January 1, 2022).

[33] R. Kronick, “Why Medicare Advantage Plans Are Being Overpaid by $200 Billion and What to Do about It,” *Health Affairs Blog*, January 29, 2020, *available at* <https://www.healthaffairs.org/do/10.1377/forefront.20200127.293799/full/> (last visited January 1, 2022); MedPAC Staff, “For the Record: MedPAC’s Response to AHIP’s Recent ‘Correcting the Record’ Blog Post,” *MedPAC Blog*, March 3, 2021, *available at* <https://www.medpac.gov/for-the-record-medpacs-response-to-ahips-recent-correcting-the-record-blog-post/> (last visited January 1, 2022); R. Agarwal , J. Connolly , S. Gupta , and A.S. Navathe , “Comparing Medicare Advantage and Traditional Medicare: A Systematic Review,” Health Affairs 40, no. 6 (2021): 937–944; A.A. Markovitz, J.Z. Ayanian, A. Warrier, and A.M. Ryan, “Medicare Advantage Plan Double Bonuses Drive Racial Disparity in Payments, Yield No Quality or Enrollment Improvements,” *Health Affairs* 40, no. 9 (2021): 1411-1419; R. Gilfillan and D.M. Berwick, “Medical Advantage, Direct Contracting, and the Medicare ‘Money Machine,’ Part 1: The Risk-Score Game,” *Health Affairs Forefront*, September 29, 2021, *available at* <https://www.healthaffairs.org/do/10.1377/forefront.20210927.6239/> (last visited July 5, 2022); Gilfillan and Berwick, *supra* note 13; P.B. Ginsburg and S.M. Lieberman, “The Debate on Overpayment in Medicare Advantage: Pulling It Together,” *Health Affairs Forefront*, February 24, 2022, *available at* <https://www.healthaffairs.org/do/10.1377/forefront.20220223.736815?vgo_ee=l8m%2FG0N1toIoheay1wFgLrTV8qsFUfI%2F1ISxnX2Ui4c%3D> (last visited July 5, 2022); Office of the Inspector General, U.S. Department of Health and Human Services, “Some Medicare Advantage Organization Denials of Prior Authorization Requests Raise Concerns About Beneficiary Access to Medically Necessary Care,” *Reports in Brief*, April 2022, OEI-09-18-00260*, available at* <https://oig.hhs.gov/oei/reports/OEI-09-18-00260.asp> (last visited July 6, 2022); N. Ochieng and J. Fuglesten Biniek, “Beneficiary Experience, Affordability, Utilization, and Quality in Medicare Advantage and Traditional Medicare: A Review of the Literature,” *Kaiser Family Foundation*, September 16, 2022, *available at* <https://www.kff.org/medicare/report/beneficiary-experience-affordability-utilization-and-quality-in-medicare-advantage-and-traditional-medicare-a-review-of-the-literature/> (last visited December 2, 2022); R. Abelson and M. Sanger-Katz, “‘The Cash Monster Was Insatiable’: How Insurers Exploited Medicare for Billions,” *New York Times Upshot*, October 8, 2022, *available at* <https://www.nytimes.com/2022/10/08/upshot/medicare-advantage-fraud-allegations.html?te=1&nl=the-morning&emc=edit_nn_20221009> (last visited December 2, 2022); United States Senate Committee on Finance, *Deceptive Marketing Practices Flourish in Medicare Advantage, available at* <https://www.finance.senate.gov/imo/media/doc/Deceptive%20Marketing%20Practices%20Flourish%20in%20Medicare%20Advantage.pdf> (last visited December 3, 2022); D.J. Meyers, A.M. Ryan, and A.N. Trivedi, “How Much of an ‘Advantage’ is Medicare Advantage?” *JAMA* 328, no. 21 (2022): 2112-2113. 36472612 10.1001/jama.2022.21892

[34] D.J. Meyers and A.N. Trivedi , “Trends in the Source of New Enrollees to Medicare Advantage From 2012 to 2019,” JAMA Health Forum 3, no. 8 (2022): e222585, *available at* <doi:10.1001/jamahealthforum.2022.2585> (last visited August 13, 2022). 36200634 PMC9375164

[35] A.J. Holmgren , D. Cutler , and A. Mehrotra , “The Increasing Role of Physician Practices as Bill Collectors Destined for Failure,” JAMA 326, no. 8 (2021): 695–696. 34328509 10.1001/jama.2021.12191

[36] R.N. D’Souza , F.S. Collins , and V.H. Murthy , “Oral Health for All — Realizing the Promise of Science,” New England Journal of Medicine 386, no. 9 (2022): 809–811; D.M. Zulman and A. Verghese, “Virtual Care, Telemedicine Visits, and Real Connection in the Era of COVID-19: Unforeseen Opportunity in the Face of Adversity,” *JAMA* 325, no. 5 (2021): 437-438; Sanders *supra* note 3; B. Sanders, “Medicare for All Act of 2022 Executive Summary,” 2022, *available at* <https//www.sanders.senate.gov/wp-content/uploads/Medicare-for-All-2022-Exec-Summary-FINAL.pdf> (last visited July 30, 2022); R. Weisman, “The Netherlands Makes Aging and Long-Term Care a Priority. In the US, It’s a Different Story,” *Boston Globe*, August 18, 2022, *available at* <https://www.bostonglobe.com/2022/08/18/world/netherlands-national-plan-makes-aging-long-term-care-priority/?s_campaign=breakingnews:newsletter> (last visited August 18, 2022); Y. Rafiei, “When Private Equity Takes Over a Nursing Home,” *The New Yorker*, August 25, 2022, *available at* <https://www.newyorker.com/news/dispatch/when-private-equity-takes-over-a-nursing-home?utm_source=nl&utm_brand=tny&utm_mailing=TNY_Daily_082522&utm_campaign=aud-dev&utm_medium=email&utm_term=tny_daily_digest&bxid=5be26599b90c2f32c60fc7cf&cndid=55384642&hasha=22c5fe33121b0022eb44296ff5714001&hashb=a3249f48d1a72ab897ae3c0e15f840ac633246fe&hashc=09b61cd023f69af2ea97c0d6e44b646878745307d0952c6d43152b5ff293e0e3&esrc=subscribe-page&mbid=CRMNYR062419> (last visited August 25, 2022); D.J. Baughman, Y. Jabbarpour, J.M. Wedstfall, A. Jetty, A. Zain, K. Baughman, B. Pollak, and A. Waheed, “Comparison of Quality Performance Measures for Patients Receiving In-Person vs Telemedicine Primary Care in a Large Integrated Health System,” *JAMA Network Open* 5, no. 9 (2022): e2233267, *available at* <https://jamanetwork.com/journals/jamanetworkopen/fullarticle/2796668> (last visited December 2, 2022); American Health Insurance Plans, “Survey of Telehealth Use by Commercial Insurance Enrollees,” December 1, 2022, *available at* <https://www.ahip.org/documents/AHIP-Telehealth-Survey-Results-12012022.pdf> (last visited December 16, 2022): J. Gerhart, A. Piff, K. Bartelt, and E. Barkley, “Telehealth Visits Unlikely To Require In-Person Follow-Up within 90 Days,” *Epic Research*, December 13, 2022, *available at* <https://epicresearch.org/articles/telehealth-visits-unlikely-to-require-in-person-follow-up-within-90-days> (last visited December 16, 2022). 35213102 10.1056/NEJMp2118478

[37] M. Johnson , S. Kishor , and D.M. Berwick , “Medicare for All: An Analysis of Key Policy Issues,” *Health Affair*s 39, no. 1 (2020): 133–141. 31905054 10.1377/hlthaff.2019.01040

[38] A. Gaffney, D. Himmelstein, and S. Woolhandler, “Congressional Budget Office Scores Medicare-For-All: Universal Coverage For Less Spending,” *Health Affairs Blog*, February 16, 2021, *available a*t **<** https://www.healthaffairs.org/do/10.1377/forefront.20210210.190243/full/ (last visited July 6, 2022); D.C. Bryant , “Single-payer Health Care: Financial Implications for a Physician,” *International Journal of Health Service*s 52, no. 3 (2022): 410–16. 35603773 10.1177/00207314221096364

[39] J. Chown, D. Dranove, C. Garthwaite, and J. Keener, “The Opportunities and Limitations of Monopsony Power in Healthcare: Evidence from the United States and Canada,” *National Bureau of Economic Research,* NBER Working Paper 26122, July 2019, *available at* <https://www.nber.org/papers/w26122> (last visited July 30 2022).

[40] M. Brenan, “Nurses Again Outpace Other Professions for Honesty, Ethics,” *Gallup Poll,* December 20, 2018*, available at* <https://news.gallup.com/poll/245597/nurses-again-outpace-professions-honesty-ethics.aspx?g_source=link_NEWSV9&g_medium=NEWSFEED&g_campaign=item_&g_content=Nurses%2520Again%2520Outpace%2520Other%2520Professions%2520for%2520Honesty%2c%2520Ethics> (last visited August 25, 2022); Airtasker, “Who Do You Trust,” May 12, 2019, *available at* <https://www.airtasker.com/blog/who-do-you-trust/> (last visited August 25, 2022); American Board of Internal Medicine, *Surveys of Trust in the U.S. Health Care System*, May 21, 2021, *available at* <https://www.norc.org/PDFs/ABIM%20Foundation/20210520_NORC_ABIM_Foundation_Trust%20in%20Healthcare_Part%201.pdf> (last visited August 25, 2022).

[41] D. Tannenbaum , C.R. Fox , and T. Rogers , “On the Misplaced Politics of Behavioural Policy Interventions,” Nature Human Behavior 1, no. 0130 (2017), *available at* < 10.1038/s41562-017-0130> (last visited August 25, 2022); Jachimowicz et al. *supra* note 14.

[42] C.S. Ho, “Medicare for All and Medicare for America: What Are We Fighting Over?” *Law and Political Economy Project Blog*, Part 1, July 23, 2019, *available at* <https://lpeproject.org/blog/medicare-for-all-and-medicare-for-america-what-are-we-fighting-over-2/>, Part 2, July 24, 2019, *available at* <https://lpeproject.org/blog/medicare-for-all-and-medicare-for-america-what-are-we-fighting-over-part-II/> (both last visited January 1, 2022); RD Williams II, “How the U.S. Can Learn from Other Countries in Reforming the Health Care System: Q & A with Thomas Rice,” *Commonwealth Fund,* June 25, 2021, *available a*t <https://www.commonwealthfund.org/blog/2021/how-us-can-learn-other-countries-reforming-health-care-system-qa-thomas-rice> (last visited January 1, 2022).

[43] V. Rodwin , “The French Health Care System,” World Hospitals and Health Services 54, no. 1 (2018): 49–55.

[44] Glied et al., *supra* note 4.

[45] Glied et al. *supra* note 4; Flood and Thomas, *supra* note 24.

[46] Flood and Thomas, *supra* note 24.

[47] R. Pollin, J. Heintz, P. Arno, J. Wicks-Lim, and M. Ash, “Economic Analysis of Medicare for All,” *Political Economy Research Institute*, November 2018, *available at* <https://peri.umass.edu/publication/item/1127-economic-analysis-of-medicare-for-all> (last visited January 1, 2022); C. Cai , J. Runte , I. Ostrer , K. Berry , N. Ponce , M. Rodriguez , S. Bertozzi , J.S. White , and J.G. Kahn , “Projected Costs of Single-Payer Healthcare Financing in the United States: A Systematic Review of Economic Analyses,” PLoS Med 17, no. 1 (2020): e1003013, *available at* <https://journals.plos.org/plosmedicine/article?id=10.1371/journal.pmed.1003013> (last visited January 1, 2022); Physicians for a National Health Plan, “Financing a Single-Payer National Health Program,” *available at* <https://pnhp.org/financing-a-single-payer-national-health-program/?eType=EmailBlastContent&eId=63611dc7-9ca4-47ef-855e-3735391a7009#projected-costs-of-single-payer-healthcare-financing-in-the-united-states-a-systematic-review-of-economic-analyses> (last visited January 1, 2022); CBOs Single-Payer Health Care Systems Team, “How CBO Analyses the Costs of Proposals for Single-Payer Health Care Systems That Are Based on Medicare’s Fee-for-Service Program,” *Congressional Budget Office*, Working Paper 2020-8, December 2020, *available at* <https://www.cbo.gov/publication/56811> (last visited July 6, 2022); Gaffney et al., *supra* note 38; J. Nelson, “Economic Effects of Five Illustrative Single-Payer Health Care Systems,” *Congressional Budget Office*, Working Paper 2022-02, February 2022, *available at* <https://www.cbo.gov/publication/57637> (last visited July 5, 2022).31940342

[48] R.H. Brook, E.B. Keeler, K.N. Lohr, J.P. Newhouse, J.E. Ware, W.H. Rogers, A. Ross Davies, C.D. Sherbourne, G.A. Goldberg, P. Camp, C. Kamberg, A. Leibowitz, J. Keesey, and D. Reboussin. “The Health Insurance Experiment: A Classic RAND Study Speaks to the Current Health Reform Debate. *RAND Corporation,* 2006, *available at* <https://www.rand.org/pubs/research_briefs/RB9174.html> (last visited January 1, 2022).

[49] A. Gaffney , S. Woolhandler , and D.U. Himmelstein . “The Effect of Large-Scale Health Coverage Expansions in Wealthy Nations on Society-Wide Healthcare Utilization,” Journal of General Internal Medicine 356, no. 8 (2019): 2406–2417; A. Gaffney, D.U. Himmelstein, S. Woolhandler, and J.G. Kahn, “Pricing Universal Health Care: How Much Would the Use of Medical Care Rise?” *Health Affairs* 40, no. 1 (2021): 105-112. 10.1377/hlthaff.2020.01715 33400569

[66] D.M. Cutler and D.P. Ly, “The (Paper)work of Medicine: Understanding International Medical Costs,” *Journal of Economic Perspectives* 25, no. 2 (2011): 3-25. 10.1257/jep.25.2.3 PMC4511963 21595323

[50] E. Gee and T. Spiro, “Excess Administrative Costs Burden the U.S. Health Care System,” *Center for American Progres*s, April 8, 2019, *available at* <https://www.americanprogress.org/issues/healthcare/reports/2019/04/08/468302/excess-administrative-costs-burden-u-s-health-care-system/> (last visited January 1, 2022); D.U. Himmelstein, T. Campbell, and S. Woolhandler, “Health Care Administrative Costs in the United States and Canada, 2017,” *Annals of Internal Medicine* 172, no. 2 (2020): 134-142; L. Tollen, E. Keating E, and A. Weil, “How Administrative Spending Contributes to Excess US Health Spending,” *Health Affairs Blog,* February 20, 2020, *available at* <https://www.healthaffairs.org/do/10.1377/hblog20200218.375060/full/> (last visited January 1, 2022); N.R. Sahni , B. Carrus , and D.M. Cutler , “Administrative Simplification and the Potential for Saving a Quarter-Trillion Dollars in Health Care,” JAMA 326, no. 17 (2021): 1677–1678; R.D. Richman, R.S. Kaplan, J. Kohli, D. Purcell, M. Shah, I. Bonfer, B. Golden, R. Hannam, W. Mitchell, D. Cehic, G. Crispin, and K.A. Schulman, “Billing And Insurance-Related Administrative Costs: A Cross-National Analysis,” *Health Affairs* 41, no. 2 (2022): 1098-1106; Health Affairs staff, “The Role of Administrative Waste in Excess US Health Spending,” *Health Affairs Research Brie*f, October 6, 2022, *available at* <https://www.healthaffairs.org/do/10.1377/hpb20220909.830296/full/> (last visited December 3, 2022).34668527

[51] P. Tseng , R.S. Kaplan , B.D. Richman , M.A. Shah , and K.A. Schulman , “Administrative Costs Associated with Physician Billing and Insurance-Related Activities at an Academic Health Care System,” JAMA 319, no. 7 (2018): 691–697; Grand View Research, “US Medical Coding Market Size, Share and Trends Analysis Report by Classification System, by Component, and Segment Forecasts, 2021-2028,” February 2021, *available at* <https://www.grandviewresearch.com/industry-analysis/us-medical-coding-market> (last visited January 1, 2022); B. Kocher and R. Rajikumar, “Setting the Stage for the Next 10 years of Health Care Payment innovation,” *JAMA* 326, no. 10 (2021); 905-906; Holmgren et al., *supra* 33; S. Howell, P.T. Yin, and J.C. Robinson, “Quantifying the Economic Burden of Drug Utilization Management on Payers, Manufacturers, Physicians, and Patients,” *Health Affairs* 40, no. 8 (2021): 1206-1214.29466590

[52] Woolhandler and Himmelstein, *supra* note 2.

[53] D. Scheinker , B.D. Richman , A. Milstein , and K.A. Schulman , “Reducing Administrative Costs in US Health Care: Assessing Single Payer and its Alternatives,” Health Services Research 56, no. 4 (2021): 615–625. 33788283 10.1111/1475-6773.13649 PMC8313956

[54] S.-Y. Kang , D. Polsky , J.B. Segal , and G.F. Anderson , “Ultra-Expensive Drugs and Medicare Part D: Spending and Beneficiary Use Up Sharply,” *Health Affair*s 40, no. 6 (2021): 1000–5; A. Gaffney and J. Lexchin, “Healing an Ailing Pharmaceutical System: Prescription for Reform for U.S and Canada,” *British Medical Journal* 361 (2018): k1039, *available at* < 10.1136/bmj.k1039> (last visited January 2, 2022); K.N. Vokinger, T.J. Hwang, P. Daniore, C.C. Lee, A. Tibau, T. Griscott, T.J. Roseman, and A.S. Kesselheim, “Analysis of Launch and Postapproval Cancer Drug Pricing, Clinical Benefit, and Policy Implications in the US and Europe,” *JAMA Oncology* 7, no. 9 (2021): e212026, *available at* <https://jamanetwork.com/journals/jamaoncology/fullarticle/2781390> (last visited January 2, 2022); P. Sorum, C. Stein, D. Wales, and D. Pratt, “A Proposal to Increase Value and Equity in the Development and Distribution of New Pharmaceuticals,” *International Journal of Health Service*s 52, no. 3 (2022): 363-371. 34097507

[55] G.C. Alexander , D.S. Knopman , S.S. Emerson , B. Ovbiagele , R.J. Kryscio , J.S. Perlmutter , and A.S. Kesselheim , “Revisiting FDA Approval of Aducanumab,” New England Journal of Medicine 385, no. 9 (2021): 769–771. 34320282 10.1056/NEJMp2110468 PMC11694499

[56] M. Ederhof, “Why We Should Standardize Provider Cost Measurement,” *Health Affairs Blog,* September 14, 2021, *available at* <https://www.healthaffairs.org/do/10.1377/hblog20210910.978390/full/> (last visited January 2, 2022).

[57] S. Kliff and J. Katz, “Hospitals and Insurers Didn’t Want You to See These Prices. Here’s Why,” *New York Times*, August 22, 2021, *available at* <https://www.nytimes.com/interactive/2021/08/22/upshot/hospital-prices.html?campaign_id=9&emc=edit_nn_20210823&instance_id=38591&nl=the-morning&regi_id=90755137&segment_id=66986&te=1&user_id=22c5fe33121b0022eb44296ff5714001> (last visited January 2, 2022); W.B. Feldman , B.N. Rome , B.L. Brown , and A.S. Kesselheim , “Payer-Specific Negotiated Prices for Prescription Drugs at Top-Performing US Hospitals,” JAMA Internal Medicine 182, no. 1 (2022): 83–86. 34747978 10.1001/jamainternmed.2021.6445 PMC8576628

[58] J.L. Billig , C.V. Kotsis , and K.C. Chung , “Trends in Funding and Acquisition of Surgical Practices by Private Equity Firms in the US from 2000 to 2020,” JAMA Surgery 156, no. 11 (2021): 1066–1068; J.M. Zhu and D. Polsky, “Private Equity and Physician Medical Practices —Navigating a Changing Ecosystem,” *New England Journal of Medicine* 384, no. 11 (2021): 981-983; J.M. Teno, “Hospice Acquisitions by Profit-Driven Private Equity Firms,” *JAMA Health Forum* 2, no. 9 (2021): e213745, *available at* <https://jamanetwork.com/journals/jama-health-forum/fullarticle/2784807> (last visited January 2, 2022); E. Fuse Brown, L. Adler, E. Duffy, P.B. Ginsburg, M. Hall, and S. Valdez, “Private Equity Investment as a Divining Rod for Market Failure: Policy Responses to Harmful Physician Practice Acquisitions,” *USC-Brookings Schaeffer Initiative for Health Policy*, October 2021, *available at* <https://www.brookings.edu/essay/private-equity-investment-as-a-divining-rod-for-market-failure-policy-responses-to-harmful-physician-practice-acquisitions/> (last visited January 2, 2022); A.C. Offodile II, M. Cerullo, M. Bindal, J.A. Rauh-Hain, and V. Ho, “Private Equity Investments in Health Care: An Overview of Hospital and Health System Leveraged Buyouts, 2003-17,” *Health Affairs* 40, no. 5 (2021): 719-26; M. Cerullo, K. Kaili Yang, J. Roberts, R.C. McDevitt, A.C. Offodile II, “Private Equity Acquisition and Responsiveness to Service-Line Profitability at Short-Term Acute Care Hospitals,” *Health Affairs* 40, no. 11 (2021):1697-1705; L.K. Olson, *Ethically Challenged: Private Equity Storms US Health Care* (Baltimore: Johns Hopkins University Press, 2022); J.B. Segal, A.P. Sen, E. Glanzberg-Krainin, and S. Hutfless, “Factors Associated with Overuse of Health Care within US Health Systems: A Cross-sectional Analysis of Medicare Beneficiaries from 2016 to 2018,” *JAMA Health Forum* 3, no. 1 (2022): e214543, *available at* <doi10.1001/jamahealthforum.2021.4543> (last visited July 3, 2022); A. La Forgia, A.M. Bond, R.T.Braun, L.Z. Yao, K. Kjaer, M. Zhang, and L.P. Casalino, “Association of Physician Management Companies and Private Equity Investment with Commercial Health Care Prices Paid to Anesthesia Practitioners,” *JAMA Internal Medi*cine 182, no. 4 (2022): 396-404; F.J. Crosson, “Physician Management Companies — Should We Care?” *JAMA Internal Medicine* 182, no. 4 (2022): 404-406; K. Weise, “Amazon to acquire One Medical clinics in latest push into health care,” *New York Times*, July 21, 2022, *available at* <https://www.nytimes.com/2022/07/21/business/amazon-one-medical-deal.html?smid=url-share&te=1&nl=the-morning&emc=edit_nn_20220722> (last visited August 1, 2022); V. Singh, Z. Song, D. Polsky, J.D. Bruch, and J.M. Zhu, “Association of Private Equity Acquisition of Physician Practices With Changes in Health Care Spending and Utilization,” *JAMA Health Forum* 3, no 9 (2022): e222886, *available a*t <doi:10.1001/jamahealthforum.2022.2886> (last visited December 2, 2022); L. Hirsch, “CVS Makes $8 Billion Bet on the Return of the House Call,” *New York Times*, September 5, 2022, *available at* <https://www.nytimes.com/2022/09/05/business/cvs-signify-health.html?te=1&nl=the-morning&emc=edit_nn_20220906> (last visited December 2, 2022); A. Kaufman, “Endgame: Heartless profiteering in the hospice industry,” *The New Yorker*, December 5, 2022: 34-43. 34431968 10.1001/jamasurg.2021.3642 PMC8387939

[59] Gillian and Berwick, *supra* notes 13 and 33; Schenker, *supra* note 53.

[60] C. Becker , “Relationships between Academic Medicine Leaders and Industry—Time for Another Look?” JAMA 324, no. 18 (2020): 1833–1814. 33170245 10.1001/jama.2020.21021

[61] M.H. Ouayogodé , A.J. Mainor , E. Meara , J.P.W. Bynum , and C.H. Colla , “Association between Care Management and Outcomes among Patients with Complex Needs in Medicare Accountable Care Organizations,” JAMA Network Open 2, no. 7 (2019): e196939, *available at* <https://jamanetwork.com/journals/jamanetworkopen/fullarticle/2737898> (last visited January 2, 2022); C.H. Colla, T. Ajayi, and A. Bitton, “Potential Adverse Financial Implications of the Merit-based Incentive Payment System for Independent and Safety Net Practices,” *JAMA* 324, no. 10 (2020): 948-50; J.M. McWilliams and A. Chen, “Understanding the Latest ACO “Savings”: Curb Your Enthusiasm and Sharpen Your Pencils — Part 1,” *Health Affairs Blog*, November 12, 2020, *available at* <https://www.healthaffairs.org/do/10.1377/hblog20201106.719550/full/> (last visited January 2, 2022); R.P. Dutton, “The Merit-Based Incentive Payment System and Quality—Is There Value in the Emperor’s new clothes?” *JAMA Network Open* 4, no. 8 (2021): e2119334, *available at* <https://jamanetwork.com/journals/jamanetworkopen/fullarticle/2782633> (last visited January 2, 2022); D. Khullar, A.M. Bond, E.M. O’Donnell, Y. Qian, D.N. Gans, and L.P. Casalino, “Time and Financial Costs for Physician Practices to Participate in the Medicare Merit-based Incentive Payment System,” *JAMA Health Forum* 2, no. 5 (2021): e210527, *available at* <https://jamanetwork.com/journals/jama-health-forum/fullarticle/2779947> (last visited January 2, 2022); J.G. Kahn and K.R. Sullivan, “Promise vs. Practice: The Actual Financial Performance of Accountable Care Organizations,” *Journal of General Internal Medicine* 37, no. 3 (2022): 680-1); A.A. Markovitz, J.M. Hollingsworth, J.Z. Ayanian, E.C. Norton, P.L Yan, and A.M. Ryan, “Performance in the Medicare Shared Savings Program after Accounting for Nonrandom Exit: An Instrumental Variable Analysis,” *Annals of Internal Medicine* 171, no. 1 (2019): 27-36; A.A. Markovitz, R.C. Murray, and A.M. Ryan, “Comprehensive Primary Care Plus Did Not Improve Quality Or Lower Spending For The Privately Insured,” *Health Affairs* 41, no. 9 (2022): 1255-62; A.M. Bond, W.L. Shapiro, L.P. Casolino, M. Zhang, and D. Khullar, “Association Between Individual Primary Care Physician Merit-based Incentive Payment System Score and Measures of Process and Payment Outcomes,” *JAMA* 328, no. 21 (2022): 2136-2146.31298714

[62] Centers for Medicare & Medicaid Services, “Affordable Care Act’s Shared Savings Program Continues to Improve Quality of Care while Saving Medicare Money during the COVID-19 Pandemic,” Press Release, August 25, 2021, *available* at <https://www.cms.gov/newsroom/press-releases/affordable-care-acts-shared-savings-program-continues-improve-quality-care-while-saving-medicarea> (last visited January 2, 2022); S. Wang, F. McStay, R.S. Saunders, D. Muhlestein, W.K. Bleser, and M.B. McClellan, “Performance Results Of The Medicare Shared Savings Program In 2011: Continued Uncertainty With Positive Movement,” *Health Affairs Forefront*, October 20, 2022, *available at* <https://www.healthaffairs.org/content/forefront/performance-results-medicare-shared-savings-program-2021-continued-uncertainty-positive> (last visited December 3, 2022).

[63] K. Yelorda , L. Rose , K. Bundorf , H.A. Muhammad , and A.M. Morris , “Association Between High-Deductible Health Plans and Hernia Acuity,” JAMA Surgery 157, no. 4 (2022): 321–326. 35152285 10.1001/jamasurg.2021.7567 PMC8842195

[64] Scott, *supra* note 5; D. Muhlestein, “Health Care Jobs Are A Political Barrier To Health Reform,” *Health Affairs Forefront*, May 25, 2022, *available at* <https://www.healthaffairs.org/do/10.1377/forefront.20220524.257311> (last visited July 7, 2022).

[65] POLITICO and Harvard Chan School, *supra* note 6; D. Himmelstein and S. Woolhandler, “Voters to Politicians: We Demand Health Reform,” *Health Justice Monitor*, December 1, 2022, *available at* <http://healthjusticemonitor.org/> (last visited December 3, 2022).

[66] V.S. Lee and B.B. Blanchfield , “Disentangling Health Care Billing for Patients’ Physical and Financial Health,” JAMA 319, no. 7 (2018): 661–3; P.J. Nelson, “Three Steps to Achieving More Affordable Health Insurance in the Individual Market,” *Health Affairs Blog*, August 19, 2021, *available at* <https://www.healthaffairs.org/do/10.1377/hblog20210813.61617/full/> (last visited January 2, 2022); M.B. Rosenthal, “The Growing Problem of Out-of-Pocket Costs and Affordability in Employer-Sponsored Insurance,” *JAMA* 326, no. 4 (2021): 305-306; A. Hoagland and P. Shafer, “Out-of-Pocket Costs for Preventive Care Persist Almost a Decade after the Affordable Care Act,” *Preventive Medicine* 150 (September 2021): 106690, abstract, *available at* < 10.1016/j.ypmed.2021.106690> (last visited January 2, 2022); S.R. Collins, D.C. Radley, and J.C. Baumgartner, “State Trends in Employer Premiums and Deductibles, 2010-2020,” *Commonwealth Fund Data Brief,* January 2022, *available at* <https://www.commonwealthfund.org/publications/fund-reports/2022/jan/state-trends-employer-premiums-deductibles-2010-2020> (last visited July 3, 2022); A.E. Carroll, “What’s Wrong with Health Insurance? Deductibles Are Ridiculous, for Starters,” *New York Times*, July 7, 2022, *available at* <https://www.nytimes.com/2022/07/07/opinion/medical-debt-health-care-cost.html> (last visited July 31, 2022). 29466571

[67] POLITICO and Harvard Chan School, *supra* note 6; A. Kirzinger, A. Kearney, M. Stokes, and M, Brodie, “KFF Health Tracking poll - May 2021: Prescription Drug Prices Top Public’s Care Priorities, *Kaiser Family Foundation*, June 3, 2021, *available at* <https://www.kff.org/health-costs/poll-finding/kff-health-tracking-poll-may-2021/> (last visited July 8, 2022).

[68] Rosenthal, *supra* note 66; Hoagland and Shafer, *supra* note 66; A. Kirzinger, C. Muñana C, B. Wu, and M. Brodie, “Data Note: Americans’ Challenges with Health Care Costs,” *Kaiser Family Foundation*, June 11, 2019, *available at* <https://www.kff.org/report-section/data-note-americans-challenges-with-health-care-costs-appendices/> (last visited January 2, 2022); C.M. Wray , M. Khare , and S. Keyhani , “Access to Care, Cost of Care, and Satisfaction with Care among Adults with Private and Public Health Insurance in the US,” JAMA Network Open 46, no. 6 (2021): e2110275, *available at* <https://jamanetwork.com/journals/jamanetworkopen/fullarticle/2780540> (last visited January 2, 2022); S.-C. Chou, A.S. Hong, G.W. Scott, and J.F. Wharam, “Impact of High-Deductible Health Plans on Emergency Department Patients with Nonspecific Chest Pain and their Subsequent Care,” *Circulation* 144, no. 5 (2021): 336-349; A. Chandra, E. Flack, and Z. Obermeyer, “The Health Costs of Cost-Sharing,” *National Board of Economic Research,* Working Paper 28439, February 2021, *available at* <http://nber.org/papers/w28439> (last visited January 2, 2022); Carroll, *supra* note 66; D. Witters, “Four in 10 Americans Cut Spending to Cover Healthcare Costs,” *Gallop Poll*, August 4, 2022, *available at* <https://news.gallup.com/poll/395126/four-americans-cut-spending-cover-healthcare-costs.aspx> (last visited August 4, 2022); S.R. Collins, L.A. Haynes, and R. Masitha, “The State of U.S. Health Insurance in 2022,” *Commonwealth Fund Issue Brief*, September 29, 2022, *available at* <https://www.commonwealthfund.org/publications/issue-briefs/2022/sep/state-us-health-insurance-2022-biennial-survey?utm_source=alert&utm_medium=email&utm_campaign=Achieving+Universal+Coverage> (last visited December 2, 2022); M. Ma, “41% of Americans with Health Insurance have Avoided Medical Care Due to Cost Factors,” *Policygenius*, October 31, 2022, *available at* <https://www.policygenius.com/health-insurance/health-insurance-survey-2022/> (last visited December 3, 2022).

[69] R. Kluender , N. Mahoney , F.D. Wong , and W. Yin , “Medical Debt in the US, 2009-2020,” JAMA 326, no. 3 (2021): 250–256; C. Mendes de Leon and J.J. Griggs, “Medical Debt as a Social Determinant of Health,” *JAMA* 326, no. 3 (2021): 328-329; L. Lopes, A. Kearney, A. Montero, L. Hamel, and M. Brodie, “Health Care Debt In the U.S.: The Broad Consequences of Medical And Dental Bills,” *Kaiser Family Foundation*, June 16, 2022, *available at* <https://www.kff.org/report-section/kff-health-care-debt-survey-appendix/> (last visited July 7, 2022).34283184

[70] M.A. Kyle and A.B. Frakt , “Patient Administrative Burden in the US Health Care System,” Health Services Research 56, no. 5 (2021): 755–765; J. Pfeffer, D. Witters, S. Agrawal, and J.K. Harter, “Magnitude and Effects of ‘Sludge’ in Benefits Administration: How Insurance Hassles Burden Workers and Cost Employers,” *Academy of Management Discoveries* 6, no. 3 (2020), abstract, *available at* <https://journals.aom.org/doi/10.5465/amd.2020.0063> (last visited January 2, 2022); J.M. Zhu, C.J. Charlesworth, D. Polsky, and K.J. McConnell, “Phantom Networks: Discrepancies Between Reported And Realized Mental Health Care Access in Oregon Medicaid,” *Health Affairs* 41, no. 7 (2022): 1013-1022; H.H. Goldman, “How Phantom Networks And Other Barriers Impede Progress On Mental Health Insurance Reform,” *Health Affairs* 41, no. 7 (2022): 1023-1025.34498259

[71] D. McCormick , S. Woolhandler , A. Bose-Kolanu , A. Germann , D.H. Bor , and D.U. Himmelstein , “U.S. Physicians’ Views on Financing Options to Expand Health Insurance Coverage: A National Survey,” Journal of General Internal Medicine 24, no. 4 (2009): 526–531; S. Kahn, J.J. Spooner, and H.E. Spotts, “United States Physician Preferences Regarding Healthcare Financing Options: A Multistate Survey,” *Pharmacy* 6, no. 4 (2018): 131, *available at* <https://www.mdpi.com/2226-4787/6/4/131> (last visited July 5, 2022); A. Abrams, “A New Generation of Activist Doctors Is Fighting for Medicare for All,” *Time*, October 24, 2019, *available a*t <https://time.com/5709017/medicare-for-all-doctor-activists/> (last visited July 5, 2022); C. Marks, “Inside the American Medical Association’s Fight Over Single-Payer Health Care,” *The New Yorker*, February 22, 2022, *available at* <https://www.newyorker.com/science/annals-of-medicine/the-fight-within-the-american-medical-association> (last visited July 5, 2022). 19184240 10.1007/s11606-009-0916-x PMC2659157

[72] Howell et al., *supra* note 51; Colla et al., *supra* note 61; P.C. Sorum , “Why Internists Might Want Single-Payer Health Care,” *Annals of Internal Medicin*e 168, no. 6 (2018): 438–439; D. Offri. “The Business of Health Care Depends on Exploiting Doctors and Nurses,” *New York Times*, June 8, 2019, *available at* <https://www.nytimes.com/2019/06/08/opinion/sunday/hospitals-doctors-nurses-burnout.html%20%E2%80%A6> (last visited January 2, 2022); D. Morra, S. Nicholson, W. Levinson, D.N. Gans, T. Hammons, and L. Casalino, “US Physician Practices versus Canadians: Spending Nearly Four Times as Much Money Interacting with Payers,” *Health Affairs* 30, no. 8 (2011): 1443-1450. 21813866 10.1377/hlthaff.2010.0893

[73] American Medical Association, “Patient Access at Risk Unless Congress Reforms Medicare Payment System,” Press Release, September 6, 2022, *available at* <https://www.ama-assn.org/press-center/press-releases/patient-access-risk-unless-congress-reforms-medicare-payment-system?&utm_source=BulletinHealthCare&utm_medium=email&utm_term=090822&utm_content=MEMBER&utm_campaign=article_alert-morning_rounds_daily&utm_uid=2384308&utm_effort=MRNRD0> (last viewed December 2, 2022); American College of Physicians, “ACP Advocacy to Prevent Physician Payment Cuts Continues,” *ACP Advocate*, December 2, 2022, *available at* <https://www.acponline.org/advocacy/acp-advocate/archive/december-2-2022/acp-advocacy-to-prevent-physician-payment-cuts-continues?utm_campaign=FY22-23_NEWS_ACPADVOCATE_120222_EML&utm_medium=email&utm_source=Eloqua> (last visited December 2, 2022).

[74] N.C. Apathy , A.J. Hare , S. Fendrich , and D.A. Cross , “Early Changes in Billing and Notes after Evaluation and Management Guideline Change,” Annals of Internal Medicine 175, no 4 (2022): 499–504; A. Gaffney, S. Woolhandler, C. Cai, D. Bor, J. Himmelstein, D. McCormick, and D.U. Himmelstein, “Medical Documentation Burden Among US Office-Based Physicians in 2019: A National Study,” *JAMA Internal Medicine* 182, no. 5 (2022): 564-6.35188791

[75] D. Blumenthal , “A Step toward Interoperability of Health IT,” New England Journal of Medicine 387, no. 24 (2022): 2201–2203. 36507704 10.1056/NEJMp2213873

[76] R. Crowley , O. Atiq , and D. Hilden , for the Health and Public Policy Committee of the American College of Physicians, “Financial Profit in Medicine: A Position Paper from the American College of Physicians,” Annals of Internal Medicine 174, no. 10 (2021): 1447–1465. 34487452 10.7326/M21-1178

[77] T.J. Hoff , Next in Line: Lower Care Expectations in the Age of Retail- and Value-Based Health (New York: Oxford University Press; 2018); J. Geyman, “The Business Ethic vs. Service Ethic in U.S. Health Care: Which will Prevail?” *The Pharos* (Winter 2022): 40-7.

[78] Pfeffer et al., *supra* note 70; S. Klein and M. Hostetter, “Tackling High Health Care Prices: A Look at Four Purchaser-Led Efforts,” *Commonwealth Fund*, April 1, 2022*, available at* <https://www.commonwealthfund.org/publications/2022/apr/tackling-high-health-care-prices-look-four-purchaser-led-efforts> (last visited July 8, 2022).

[79] 111th Congress, “Patient Protection and Affordable Care Act,” Public Law 111-148, *available a*t <https://www.congress.gov/111/plaws/publ148/PLAW-111publ148.pdf> (last visited July 7, 2022).

[80] P.J. Neumann , A.B. Rosen , and M.C. Weinstein , “Medicare and Cost-Effectiveness Analysis,” New England Journal of Medicine 353, no. 14 (2005): 1516–1522; P.J. Neumann, “American Exceptionalism and American Health Care: Implications for the US Debate on Cost-Effectiveness Analysis,” *Office of Health Economics*, OHE Briefing no. 47 (2009); J.D. Chambers, S. Morris, P.J. Neumann, and M.J. Buxton, “Factors Predicting Medicare National Coverage: An Empirical Analysis,” *Medical Care* 50, no. 3 (2012): 249-56; J.D. Chambers, M.J. Cangelosi, and P.J. Neumann, “Medicare’s Use of Cost-Effectiveness Analysis for Prevention (but not for Treatment),” *Health Policy* 119, no. 2 (2015): 156-63.16207857

[81] 111th Congress, supra note 79, Sec 1182(e), p. 742.

[82] H.A Glick . S. McElligott , M.V. Pauly , R.J. Willke , J. Bergquist , J. Doshi , L.A. Fleisher , B. Kinosian , E. Perfetto , D.E. Polsky , and J.S. Schwartz , “Comparative Effectiveness and Cost-Effectiveness Analyses Frequently Agree On Value *,”* Health Affairs 34, no. 5 (2015): 805–811; G. Persad, “Priority Setting, Cost-Effectiveness, and the Affordable Care Act,” *American Journal of Law & Medicine* 41, no. 1 (2015): 119-66. 25941282 10.1377/hlthaff.2014.0552

[83] T.S. Jost , “The Independent Payment Advisory Board,” New England Journal of Medicine 363, no. 2 (2010): 103–5. 20505171 10.1056/NEJMp1005402

[84] J.E. McDonough , “In Defense of the Independent Payment Advisory Board,” Milbank Quarterly 95, no. 3 (2017): 466–469. 28895224 10.1111/1468-0009.12271 PMC5594281

[85] M. K. Gusmano , M. Laugesen , V. G. Rodwin , and L. D. Brown , “Getting the Price Right: How Some Countries Control Spending in a Fee-for-Service System,” Health Affairs 39, no. 11 (2020): 1867–74. 33136495 10.1377/hlthaff.2019.01804

[86] Kocker and Rajikumar, *supra* note 51; J. Adler-Milstein and A. Mehrotra , “Paying for Digital Health Care—Problems with the Fee-for-Service System,” New England Journal of Medicine 385, no. 10 (2021): 871–3; J. Kahn, “Fee-for-Service vs. Capitation,” *Health Justice Monitor*, March 3, 2022, *available at* <http://healthjusticemonitor.org/2022/03/03/fee-for-service-vs-capitation/> (last visited July 5, 2022); B.E. Outland, S. Erickson, R. Doherty, W. Fox, and L. Ward, “Reforming Physician Payments to Achieve Greater Equity and Value in Health Care: A Position Paper of the American College of Physicians,” *Annals of Internal Medicine* 175, no. 7 (2022): 1019-21. 35724380 10.7326/M21-4484

[87] Tikkanen et al., *supra* note 4: at 227; R. A. Berenson and R. B. Murray , “How Price Regulation Is Needed to Advance Market Competition,” Health Affairs 41, no. 1 (2022): 26–34: Emanuel et al., *supra* note 4; D.W. Johnson, M. Guido, and M. Goozner, “Meaningful Value-Based Payment Reform, Part I: Maryland Leads The Way,” *Health Affairs Forefront*, February 9, 2022, *available at* <https://www.healthaffairs.org/do/10.1377/forefront.20220205.211264> (last visited July 7, 2022); R. Murray, “Hospital Global Budgets: A Promising State Tool for Controlling Health Care Spending,” *Commonwealth Fun*d *Issue Brief*, March 22, 2022, *available at* <https://www.commonwealthfund.org/publications/issue-briefs/2022/mar/hospital-global-budgets-state-tool-controlling-spending> (last visited July 7, 2022); Brennan, *supra* note 10. 34982623 10.1377/hlthaff.2021.01235

[88] T. Bodenheimer and C. Sinsky , “From Triple to Quadruple Aim: Care of the Patient Requires Care of the Provider,” Annals of Family Medicine 12, no. 6 (2014): 573–576; Dutton, *supra* note 61; L. Rosenbaum, “Reassessing Quality Assessment—The Flawed System for Fixing a Flawed System,” *New England Journal of Medicine* 386, no. 17 (2022); 1663-1667; W.V. Padula and P.J. Provonost, “Value Defects In The Health Services Sector,” *Health Affairs Forefront*, May 4, 2022, *available a*t <https://www.healthaffairs.org/do/10.1377/forefront.20220502.964577> (last visited July 7, 2022); A. Malinow, “REACH Won’t Fix the Equity Harms of Value-Based Care,” *Health Justice Monitor,* July 13, 2022, *available at* <http://healthjusticemonitor.org/> (last visited August 1, 2022). 25384822 10.1370/afm.1713 PMC4226781

[89] L.F MacMahon , K. Rize , N, Irby-Johnson , and V. Chopra , “Designed to Fail? the Future of Primary Care,” Journal of General Internal Medicine 36, no. 2 (2021): 515–517; J.H. Wasson, H.C. Sox, and H.D. Miller, “Aligning Payments, Services, and Quality in Primary Care,” *JAMA* 326, no. 9 (2021): 805-806; K. Grumbach, T. Bodenheimer, D. Cohen, R.L. Phillips, K.C. Stange, and J.M. Westfall, “Revitalizing the U.S. Primary Care Infrastructure,” *New England Journal of Medicine* 385, no. 13 (2021): 1156-8; L. McCauley, R.L. Philips, Jr., M. Meisnere, and S. Robinson, eds., *Implementing High-Quality Primary Care: Rebuilding the Foundation of Health Care (*Washington, DC: The National Academies Press; 2021, *available at* <http://nap.edu/25983> (last visited July 5, 2022); E. Rourke, “In Clinical Care, What Will Amazon Deliver?” *New England Journal of Medicine* 385, no. 26 (2021): 2401-3; D. Blumenthal, “Can New Players Revive U.S. Primary Care?” *Harvard Business Review*, January 7, 2022, *available at* <https://hbr.org/2022/01/can-new-players-revive-u-s-primary-care> (last visited July 4, 2022); J.S. Galea, “The Post-COVID-19 Case for Primary Care,” *JAMA Health Forum* 3, no. 7 (2022): e223096, *available at* <https://jamanetwork.com/journals/jama-health-forum/fullarticle/2794949> (last visited August 1, 2022); D. Blumenthal and L. Gustafsson, “Amazon’s Foray into Primary Care Won’t Be Easy,” *Harvard Business Review*, August 2, 2022, *available at* <https://hbr.org/2022/08/amazons-foray-into-primary-care-wont-be-easy> (last visited August 19, 2022); L.S. Hughes, D.J, Cohen, and R.L. Phillips Jr., “Strengthening Primary Care to Improve Health Outcomes in the US,” *JAMA Health Forum* 3, no. 9 (2022): e22903, *available at* <https://jamanetwork.com/journals/jama-health-forum/fullarticle/2795943> (last visited December 2, 2022); Primary Care Collaborative, “Relationships Matter: How Usual is Usual Source of (Primary) Care? 2022 PCC Evidence Report,” November 16, 2022, *available at* <https://www.pcpcc.org/sites/default/files/resources/pcc-evidence-report-2022_1.pdf> (last visited December 3, 2022); S. Shah, H. Rooke-Ley, and E.C Fuse Brown, “Corporate Investors in Primary Care—Profits, Progress, and Pitfalls,” *New England Journal of Medicine* 388, no. 2 (2023): 99-101. 36617356 10.1056/NEJMp2212841

[90] B.J. Miller, J. Slota, T. Cullen, G. Lushniak, and G.R. Wilensky, “To Transform Veterans Health Care for the Next Generation, We Should Learn from TRICARE,” *Health Affairs Blog,* July 26, 2021, *available at* <www.healthaffairs.org/do/10.1377/hblog20210721.600774/full/> (last visited January 2, 2022).

[91] M. Jewers and L. Ku , “Noncitizen Children Face Higher Health Harms Compared with their Siblings Who Have US Citizen Status,” Health Affairs 40, no. 7 (2021): 1984–9. 34228524 10.1377/hlthaff.2021.00065

[92] D.U. Himmelstein, S. Woolhandler, A. Gafney, D. McCanne, and J. Geyman, “Medicare for All Is Not Enough,” *The Nation*, March 31, 2022, *available at* <https://www.thenation.com/article/economy/healthcare-corporations-private-equity/> (last visited July 6, 2022); Physicians for a National Health Program, “Understanding the Medicare for All Act of 2022,” *available at* <https://pnhp.org/what-is-single-payer/senate-bill/?eType=EmailBlastContent&eId=8ab50f8a-a0c7-4dfa-a669-ad4ddc22380f > (last visited August 14, 2022); V. Curto, A.D. Sinaiko, and M.B. Rosenthal, “Price Effects of Vertical Integration and Joint Contracting between Physicians and Hospitals in Massachusetts,” *Health Affairs* 41, no. 5 (2022): 741-50. 10.1377/hlthaff.2021.00727 PMC10249654 35500187

[93] M. Nguyen , S.J. Chaudhry , M.M. Desai , C. Chen , H.R.C. Mason , W.A. McDade , T.L. Fancher , and D. Boatright , “Association of Sociodemographic Characteristics With US Medical Student Attrition,” JAMA Internal Medicine 182, no. 9 (2022): 917–24; M. Tobey, A. Ott, and M. Owen, “The Indian Health Service and the Need for Resources to Implement Graduate Medical Education Programs,” *JAMA* 328, no. 4: 327-8. 35816334 10.1001/jamainternmed.2022.2194 PMC9274446

[94] Thaler and Sunstein, *supra* note 14.

[95] E. Klein, “It’s Time to Move past Employer-Based Health Insurance,” *Vox*, April 9, 2020, *available at* <https://www.vox.com/2020/4/9/21210353/coronavirus-health-insurance-biden-sanders-medicare-for-all> (last visited January 2, 2022); M.K. Bundorf , S. Gupta , and C. Kim C, “Trends in US Health Insurance Coverage during the COVID-19 Pandemic,” JAMA Health Forum 2, no. 9 (2021): e212487 *, available at* <https://jamanetwork.com/journals/jama-health-forum/fullarticle/2783874> (last visited January 2, 2022). 35977184 10.1001/jamahealthforum.2021.2487 PMC8796896

[96] Byyny, 2020, *supra* note 27; P. Yerramilli , F.P. May , and V.B. Kerry , “Reducing Health Disparities Requires Financing People-Centered Primary Care,” JAMA Health Forum 2, no. 2 (2021): e 201573, *available at* <https://jamanetwork.com/journals/jama-health-forum/fullarticle/2776056> (last visited January 2, 2022); L. Hiam and R. Yates, “Will the COVID-19 Crisis Catalyze Universal Health Reforms?” *Lancet* 398, no. 10301 (2021): 646-648; T. Campbell, A.P. Galvani, G. Friedman, and M.C. Fitzpatrick, “Exacerbation of COVID-19 Mortality by the Fragmented United States Healthcare System: A Retrospective Observational Study,” *Lancet Regional Health – Americas* 12: 100264 (2022), *available at* <https://www.sciencedirect.com/science/article/pii/S2667193X22000813?via%3Dihub> (last visited July 7, 2022); A.P. Galvani, A.S. Parpia, A. Pandey, P. Sah, K. Colón, G. Friedman, T. Campbell, J.G. Kahn, B.H. Singer, and M.C. Fitzgerald, “Universal Healthcare as Pandemic Preparedness: The Lives and Costs that Could have been Saved During the COVID-19 Pandemic,” *PNAS* 119, no. 25 (2022): e2200536119, *available at* <https://www.pnas.org/doi/full/10.1073/pnas.2200536119> (last visited July 7, 2022); National Academy of Medicine, *Emerging Stronger from COVID-19: Priorities for Health System Transformation* (Washington, DC: National Academies Press, 2022); E.C. Fuse Brown and A.S. Kesselheim, “The History of Health Law in the United States,” *New England Journal of Medicine* 387, no. 4 (2022): 289-292; S. Corlette and C.H. Monahan, “U.S Health Insurance Coverage and Financing,” *New England Journal of Medicine* 387, no. 25 (2022): 2297-2300. 10.1001/jamahealthforum.2020.1573 36218782

